# Oncogene Concatenated Enriched Amplicon Nanopore Sequencing for rapid, accurate, and affordable somatic mutation detection

**DOI:** 10.1186/s13059-021-02449-1

**Published:** 2021-09-06

**Authors:** Deepak Thirunavukarasu, Lauren Y. Cheng, Ping Song, Sherry X. Chen, Mitesh J. Borad, Lawrence Kwong, Phillip James, Daniel J. Turner, David Yu Zhang

**Affiliations:** 1 NuProbe USA Inc, Houston, TX USA; 2 Department of Bioengineering, Rice University, Houston, TX USA; 3 Department of Oncology, Mayo Clinic, Phoenix, AZ USA; 4 Department of Translational Molecular Pathology, The University of Texas MD Anderson Cancer Center, Houston, TX USA; 5 Oxford Nanopore Technologies, Oxford, UK

**Keywords:** Nanopore Sequencing, Cancer, Mutation detection

## Abstract

We develop the Oncogene Concatenated Enriched Amplicon Nanopore Sequencing (OCEANS) method, in which variants with low variant allele frequency (VAFs) are amplified and subsequently concatenated for Nanopore Sequencing. OCEANS allows accurate detection of somatic mutations with VAF limits of detection between 0.05 and 1%. We construct 4 distinct multi-gene OCEANS panels targeting recurrent mutations in acute myeloid leukemia, melanoma, non-small- cell lung cancer, and hepatocellular carcinoma and validate them on clinical samples. By demonstrating detection of low VAF single nucleotide variant mutations using Nanopore Sequencing, OCEANS is poised to enable same-day clinical sequencing panels.

**Supplementary Information:**

The online version contains supplementary material available at (10.1186/s13059-021-02449-1).

## Background

High-throughput DNA sequencing is becoming a standard part of oncology care, with many laboratory-developed tests informing patient prognosis [1], therapy selection[2, 3], and minimal residual disease [4]. DNA sequencing is furthermore being explored as a method for enabling early screening of cancers in asymptomatic populations [5, 6]. The dominant platforms used for clinical high-throughput sequencing today are based on sequencing-by-synthesis (next-generation sequencing (NGS), e.g., the Illumina and Ion Torrent platforms).

Although NGS has many advantages including high throughput, high sensitivity, and high reproducibility/reliability, NGS also has three notable limitations: First, NGS read lengths are limited to roughly 300 nt, rendering it less suitable/sensitive to larger scale DNA alterations such as copy number variations and chromosomal translocations. Second, NGS is time-consuming, with multiple days needed for sequencing-by-synthesis chemistry, in addition to time- and labor-intensive library preparation and bioinformatic interpretation. Third, NGS requires a high capital investment on the order of <DOLLAR/>1,000,000 for a high-throughput instrument (e.g., Novaseq); more affordable NGS platforms such as the MiniSeq result in roughly 10-fold higher per-read sequencing costs.

Nanopore Sequencing overcomes all three of the above limitations of NGS, with reads as long as the fragments that are loaded, sequencing times as short as 15 min, and Oxford Nanopore MinION instrument plus starter pack costing approximately <DOLLAR/>1000 and size of a USB drive. On the other hand, while Nanopore Sequencing has historically had a higher error rate than NGS, there has been intense recent research effort in reducing Nanopore Sequencing error rates on both the hardware [7] and software [8] sides, which has brought the raw read error rate up to a competitive level [7, 9]. Detection of somatic single-base mutations at low variant allele frequencies (VAF) has not been demonstrated in peer reviewed publications [10–12]. New technologies are starting to be developed to enable low VAF detection using Nanopore Sequencing [35]. However, rapid mutation profiling with turn around times less than 1 day has not been demonstrated.

The importance of structural variations in cancer are beginning to be understood using long-read technologies. However, a large fraction, if not a majority, of actionable cancer DNA alterations reported to date were done so using short-read technologies and are therefore are single-base mutations [13], and in tumor tissue samples, there may be significant cancer heterogeneity and/or low tumor fraction. Consequently, reliable detection of single-base mutations at 5% VAF in formalin-fixed, paraffin-embedded (FFPE) tissues is currently considered a standard requirement for clinical NGS assays [14, 28]. Cell-free DNA in peripheral blood plasma is an emerging biospecimen for noninvasive cancer monitoring [15] and has even more stringent requirements on VAF limit of detection (LoD), with typical commercial NGS kits and services claiming LoDs of between 0.1 and 0.5% VAF [29, 30].

**Fig. 1:**
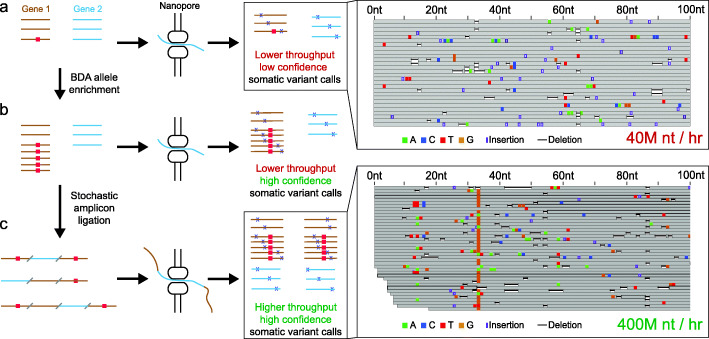
Overview of Oncogene Concatenated Enriched Amplicon Nanopore Sequencing (OCEANS) approach. (a) Short DNA amplicons from genes or loci of interest potentially bearing somatic mutations (red rectangle) have a lower throughput and low confidence somatic variant calls without using OCEANS. (b) We first use the blocker displacement amplification (BDA) technology to selectively amplify DNA sequence variants, so that somatic mutations with low sample VAF (≤5*%*) are represented in high VAF in the prepared DNA library. (c) Subsequently, we enzymatically concatenate the amplicons to increase the effective throughput of Nanopore Sequencing. OCEANS exhibits roughly 100-fold better mutation VAF limit of detection and roughly 10-fold higher throughput compared to the short amplicon SQK-LSK109 Nanopore dataset generated without using OCEANS

Here, we present a new method, Oncogene Concatenated Enriched Amplicon Nanopore Sequencing (OCEANS), that achieves ≤1% VAF LoD on single-base mutations in FFPE samples by Nanopore Sequencing (Fig. 1). First, we describe a amplicon concatenation method called Stochastic Amplicon Ligation (SAL) to utilize the long read capabilities of Nanopore Sequencing for sequencing short amplicons. SAL approach would be suitable for any amplicon-based short fragment library from samples like FFPE and cfDNA. The OCEANS method integrates the blocker displacement amplification (BDA) allele enrichment method [16, 17] with SAL, improving the VAF LoD by roughly 100-fold and throughput by roughly 10-fold using SQK-LSK109 sample prep chemistry on R9.4.1 flow cells. The entire OCEANS method takes less than 10 h from DNA to called variants, and the average sequencing cost per FFPE sample is roughly <DOLLAR/>7.5 for a 7-gene, 15-amplicon panel on the Oxford Nanopore MinION flow cells.

## Results

**Stochastic Amplicon Ligation.** DNA samples for oncology sequencing are typically extracted from FFPE tissues and can have average lengths of less than 500 nt due to accumulated chemical damage [18]. We developed the Stochastic Amplicon Ligation (SAL) method to enzymatically concatenate many short DNA molecules together to utilize the long-read capability of Nanopore Sequencing and increase the effective throughput.

**Fig. 2:**
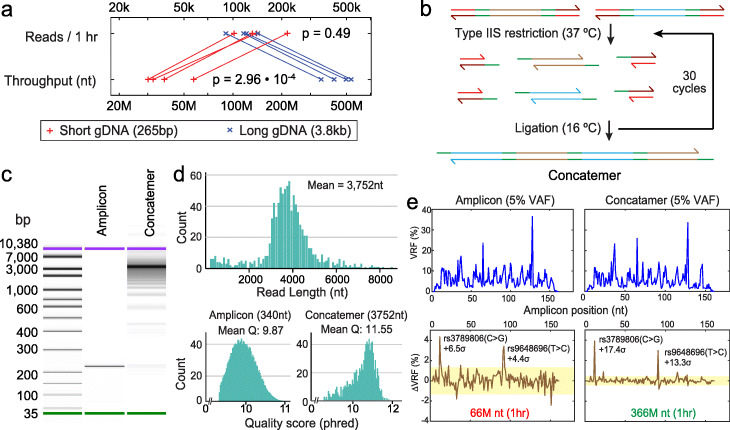
Concatenation of short amplicons by Stochastic Amplicon Ligation (SAL). **a** The number of Nanopore Sequencing reads per hour observed for two different lengths of DNA using SQK-LSK109 sample prep chemistry on R9.4.1 flow cells. Here, we performed Nanopore Sequencing on the NA18562 human genomic DNA (gDNA) physically sheared (Covaris g-tube) to an average of 265 base pairs (bp) or 3800 bp (3.8 kb). The effective throughput of the longer gDNA library was roughly 10-fold higher than the shorter gDNA library. **b** Concatenation of DNA amplicons through SAL. Amplicons are adapted so that their 5 ^′^ and 3 ^′^ ends possess Type IIS restriction enzyme sites. Through a series of restriction and ligation reactions, >10 amplicons are assembled into concatemers. Compared to a naive method based on blunt end ligation, SAL enriches longer concatemers by maintaining low concentrations of amplicon monomers during each cycle of the reaction. Additionally, SAL significantly increases the on-target rate of the final Nanopore Sequencing libraries by excluding undesired dsDNA molecules from being incorporated into the concatemer. **c** Capillary electrophoresis analysis of a 220 nt amplicon and its SAL concatemer products. **d** Nanopore Sequencing read lengths of concatemers for a 7-plex SAL reaction from amplicons with a mean length of 340 nt. In addition to the increased throughput of Nanopore Sequencing for the concatemer due to the longer DNA lengths, we also observed a significant increase in sequencing quality for the concatemer vs. the original amplicon. **e** Increased Nanopore Sequencing throughput from SAL improves the limit of detection of somatic mutations when paired normal samples are available. The top traces show the variant read fraction (VRF) of Nanopore Sequencing reads at each location that corresponds to the highest frequency single-base changes (substitution, insertion deletion). The bottom traces show the relative excess of the top variant at each position for the 5% VAF samples compared to a 0% VAF sample; see Additional file 1: Fig. S7 for amplicon Nanopore Sequencing traces for 0% VAF samples. The two SNP alleles specific to NA18562 were more prominently called in the SAL concatemer Nanopore Sequencing results. The input DNA for each run was either 50 ng of a 95%:5% mixture of the NA18537 and NA18562 cell line human genomic DNA (gDNA), or 50 ng NA18537. Note: The results in panels a, d and e are specifically using SQK-LSK109 sample prep chemistry on R9.4.1 flow cells using MinKNOW 19.12.5 for basecalling

SAL is based on the Golden Gate assembly method used in synthetic biology to concatenate short oligos into synthetic genes [19]. In SAL (Fig. 2 b), amplicons are appended with engineered adapter sequences that possess a Type IIS restriction enzyme recognition site. After Type IIS cleavage, a 4 nt sticky end is left on the 5 ^′^ ends of both strands of the amplicons; these sticky ends allow the amplicons to transiently bind to each other, which then enzymatically ligate to form concatemers. Multiple cycles of enzymatic restriction and ligation are performed to increase the lengths of the concatemers, and we perform a SPRI (solid phase reversible immobilization) size selection afterwards to both enrich long concatemers and remove short recognition site oligos cleaved from the amplicons. The multiple temperature cycles between 37 and 16 ^∘^C improve the mean lengths of the concatemer assemblies by keeping the concentrations of activated monomer amplicons low. Prior literature [19] suggests that direct ligation of amplicons with 5 ^′^ sticky ends results in a much larger population of shorter concatemers.

SAL differs from traditional Golden Gate assembly in having universal sticky end sequences to allow stochastic incorporation of any amplicon with the appropriate adapters. This, in principle, allows unlimited growth of concatemers to longer lengths given sufficient monomer concentration and enough temperature cycles and reduces the possibility of unintended reactions due to nonspecific binding between non-cognate sticky ends. Experimentally, capillary electrophoresis indicated that SAL concatenated a mean of roughly 12 to 15 monomers per concatemer (Fig. 2 c). In addition to improving the throughput of Nanopore Sequencing, we also found that the SAL improved the quality of Nanopore Sequencing reads (Fig. 2 d). The Nanopore Sequencing results of a 340-nt amplicon had a mean phred quality score of 9.87, corresponding to an error rate of 10.3%. The concatemer, in contrast, had a mean phred score of 11.55, corresponding to an error rate of 7.0%. The lower quality score of shorter reads is due to lack of sufficient current signal information for proper normalization prior to basecalling by MinKNOW (Personal communication by ONT technical support).

The improved throughput and accuracy of Nanopore Sequencing of SAL concatemers allow calling somatic single-base mutations at 5% VAF when matched normal sample are available (Fig. 2 e). We first applied Nanopore Sequencing to amplicons from a 95%:5% mixture of the NA18537 and NA18562 human cell line genomic DNA. NA18537 and NA18562 were homozygous for different alleles at the rs3789806 and rs9648696 single nucleotide polymorphism (SNP) loci, so the mixture was 5% VAF in the NA18537 SNP alleles. In our analysis, it was difficult to call somatic mutations at 5% VAF, since there was a large number of loci on the amplicon with high variant read frequencies. When we subtracted the variant read fraction (VRF) from a 100% NA18537 sample, then the 5% VAF somatic mutations became more visible in the *Δ*VRF. In direct amplicon nanopore sequencing, the two 5% VAF SNP alleles were detected in the *Δ*VRF figure with +6.5 *σ* and +4.4 *σ*, respectively. The confidence of calling these 5% VAF variants were increased to +17.4 *σ* and +13.3 *σ* for SAL concatemers. For SAL concatemers, the long nanopore sequencing reads were bioinformatically deconcatenated using a custom python code [33].

The lower throughput and quality score of shorter DNA libraries are specific to SQK-LSK109 sample prep chemistry on R9.4.1 flow cells using MinKNOW 19.12.5 for basecalling. Sequencing throughput and quality of short libraries could be on par with long DNA libraries using ONT’s latest and upcoming improvements to sequencing chemistry and basecalling algorithms [36].

Rolling circle amplification (RCA) is an alternative method for generating long DNA from shorter DNA molecules, which has been used in the context of Nanopore Sequencing for improving accuracy [20]. RCA has several significant limitations compared to SAL, most notably that RCA requires an initial circularization of DNA which is known to have low efficiency, reducing the clinical sensitivity due to low conversion yield of biological DNA molecules in sequencing library. Additionally, RCA produces concatemers in which the segment sequences all reflect the sequence of the original molecule, rather than a uniform sampling of all DNA molecules on the loci of interest. Finally, RCA generates single-stranded DNA products rather than double-stranded DNA products, which should be converted into double-stranded DNA for efficient Nanopore Sequencing.

**Integrating BDA allele enrichment.** Frequently, matched normal FFPE tissue samples will not be available, so using SAL alone for Nanopore Sequencing detection of low VAF somatic mutations is unlikely to be impactful clinically. The OCEANS method employs blocker displacement amplification (BDA) [16, 17] to allow more robust detection of low VAF somatic mutations without requiring a matched normal sample. In brief, BDA includes a wildtype-binding blocker oligonucleotide that competes with a PCR primer in hybridizing to DNA templates of interest. The blocker binds more strongly than the primer to wildtype DNA sequences, preventing efficient PCR amplification. On DNA templates with sequence variants, the primer outcompetes the blocker and PCR proceeds as usual. Through the course of many PCR cycles (20–25 cycles), the VAF of sequence variants (including single nucleotide mutations) can be enriched by over 1000-fold.

**Fig. 3:**
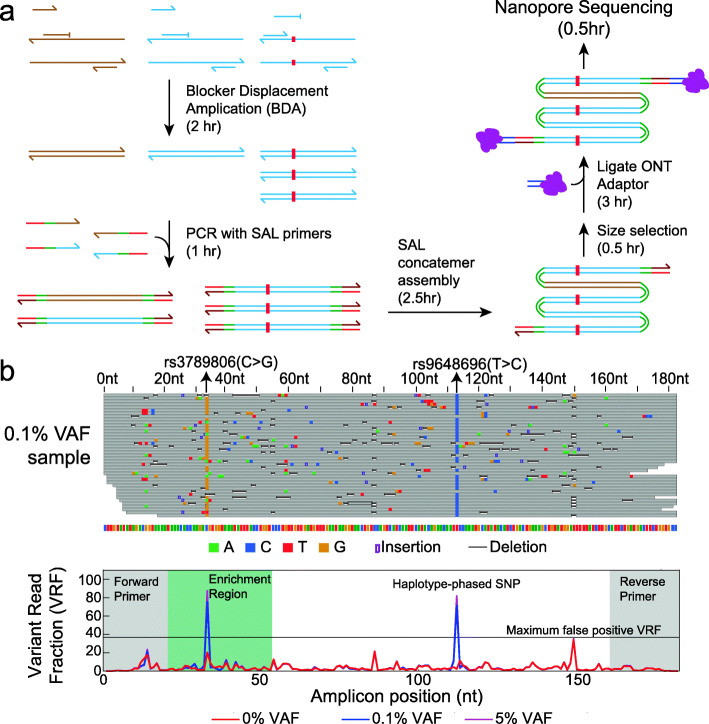
Method and experimental results for the full OCEANS method. **a** Potential variants in multiple genetic loci of interest are first enriched using multiplex blocker displacement amplification (BDA) [16, 17]. The amplicons are subsequently appended with SAL adapters using PCR and assembled into concatemers. After size selection to remove short concatemers and excess primers, the concatemers are ligated to the Oxford Nanopore adapter bearing a motor protein, and sequenced using the MinION platform. **b** OCEANS enables confident variant calls of single-base variants at 0.1% VAF without a matched normal sample. The top diagram shows a randomly selected subset of Nanopore Sequencing reads, and the bottom diagram shows the variant read frequency (VRF), the fraction of Nanopore Sequencing reads at each locus that corresponds to the most frequent single-base variant. The forward and reverse primer regions are shaded in gray, and the BDA enrichment region is shaded in green

In OCEANS, the DNA biospecimen is first mixed with multiple primers and blockers to undergo variant-selective PCR amplification (Fig. 3 a). The amplicons will over-represent sequence variants in genetic loci of interest, though some amplicons with wildtype sequences will still exist. The amplicons are subsequently appended with SAL adapters and concatenated into concatemers, and size-selected to remove short assemblies, primers, etc. The concatemers are then ligated to the standard Oxford Nanopore Sequencing adapters with attached motor proteins and loaded into the nanopore sequencing flow cell. The entire workflow takes about 10 h, including post-sequencing bioinformatics. On the same SNP alleles as in Fig. 2, the OCEANS results showed VRFs that were dramatically higher than the sample variant VAFs (Fig. 3 b), with 0.1% VAF SNP allele enriched to over 70% VRF. Thus, OCEANS allows robust variant calls of somatic mutations without the need for a matched normal DNA sample, which was not possible previously on the Nanopore Sequencing platform.

**Fig. 4:**
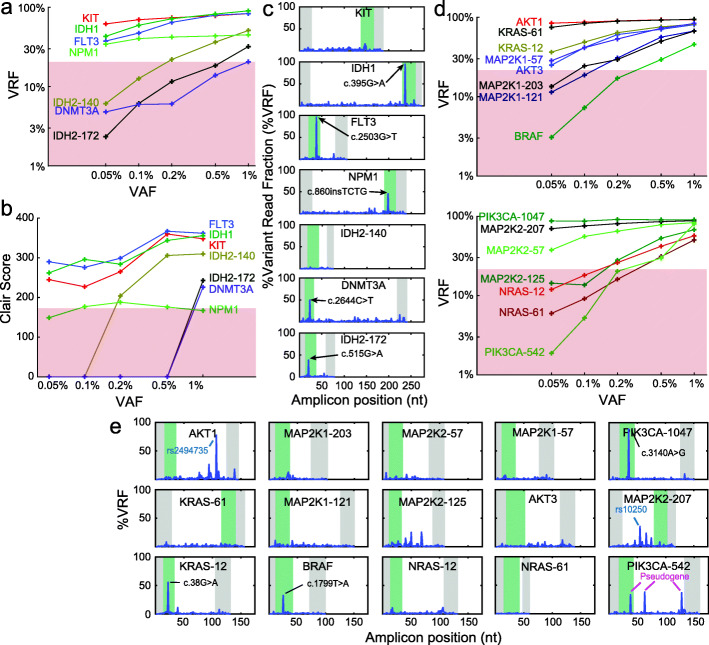
Bioinformatics and limit of detection of somatic mutation calls from multi-gene OCEANS panels. **a** Summary of variant read frequencies (VRF) observed for a 7-plex OCEANS panel covering recurrent mutations in the KIT, IDH1, FLT3, NPM1, IDH2, and DNMT3A genes, which are present in high frequency in acute myeloid leukemia (AML). The input samples used here were internal reference samples constructed by spiking known quantities of synthetic DNA bearing a mutation of interest into NA18562 gDNA; see Adiitional file 1: Section S4 for summary of Nanopore Sequencing results on these experiments. Based on calibration experiments, we tentatively propose a 20% VRF cutoff for calling a variant. The limit of detection for different mutations varied between ≤0.05% VAF (for KIT, IDH1, FLT3, and NPM1) and 1% VAF (for DNMT3A). **b** Qualitative de novo variant calls using the Clair [21] bioinformatics pipeline, on the same data as in panel (a). Based on Oxford Nanopore internal calibration, Clair scores ≥180 are reliable. Although the Clair variant call differed from the VRF-based variant call for the NPM1 mutation, all other mutations were detected with similar VAF limits of detection. To be conservative and reduce false positive variant calls, we typically make a somatic variant call only when both VRF ≥20% and Clair ≥180. **c** Results of the AML 7-plex OCEANS panel on 50ng of a third-party reference DNA sample (Horizon HD829). All mutations in the reference sample covered by our panel were at 5% VAF, and were detected. **d** Summary of VRF observed for different internal reference samples using a 15-plex OCEANS panel covering 7 genes frequently mutated in melanoma (AKT1, AKT3, KRAS, MAP2K1, MAP2K2, NRAS, and PIK3CA). For this panel, the limits of detection varied between ≤0.05% VAF and 0.5% VAF. **e** Results of the melanoma 15-plex OCEANS panel on 50ng of a 99%:1% mixture of NA18562 gDNA and a third-party reference DNA sample (Horizon HD238). The HD238 sample is nominally a reference sample that is 50% VAF in BRAF-V600E (c. 1799T >A), so the input sample should be positive only for BRAF-V600E at 0.5% VAF. Upon our discovery of the KRAS-c.38G >A and PIK3CA-c.3140A >G mutations, we checked with the manufacturer and confirmed that these mutations are also present in the reference sample at low VAFs

We next constructed two multiplexed OCEANS panels: a 7-amplicon panel covering recurrent mutations observed in acute myeloid leukemia (AML) and a 15-amplicon panel covering recurrent mutations observed in melanoma. The AML panel covers roughly 254 mutations in the COSMIC database across 7 genes (Fig. 4 a), and the melanoma panel covers roughly 370 mutations across 8 genes (Fig. 4 d). We first characterized the limit of detection for mutations covered by these OCEANS panels using synthetic spike-in reference samples, with VAFs ranging from 0.05 and 1%.

Variant calls were made using two different approaches: (1) based on the variant read frequency exceeding a threshold of 20% and (2) based on a Clair [21] score of above 180 (Personal communication from ONT). We found that both approaches were imperfect: considering VRF alone ignores the fact that Nanopore Sequencing has different error rates for certain sequences, e.g., homopolymers. Clair scores, on the other hand, are not monotonic with VAF and have been observed to be less accurate for indel calls [10, 25–27]. To ensure minimal false positives in variant calls, we require that a variant must be independently called by both methods in order to be reported. On our internal reference samples (Fig. 4 a, b, d), we observed VAF limits of detection between 0.05 and 1%. We did not observe any effect on enrichment of variants by excluding SAL from OCEANS workflow (Additional file 1: Figure S11). But SAL significantly increases the throughput for short amplicons by harnessing the long read capabilities of Nanopore Sequencing. We next applied our OCEANS panels to third-party reference samples from Horizon Discovery, with mutation VAFs at 5% (for AML) and 0.5% (for melanoma). The expected mutations were all called.

Interestingly, we also made a number of unexpected variant calls in the melanoma OCEANS panel (Fig. 4 e). The variant in the MAP2K2-207 amplicon was confirmed to be a non-pathogenic SNP (rs10250). The variants in the PIK3CA-542 amplicon were found to be aligned to the PIK3CA pseudogene (LOC100422375) [22], which were also preferentially enriched by BDA. The “variants” associated with the PIK3CA pseudogene were bioinformatically excluded from variant calls in subsequent experiments. Finally, we made confident variant calls for PIK3CA c.3140A >G and KRAS c.38G >A. We contacted Horizon Discovery customer support regarding these putative mutations, and the latter confirmed that these mutations are also present at low VAFs in the HD238 sample.

**Fig. 5:**
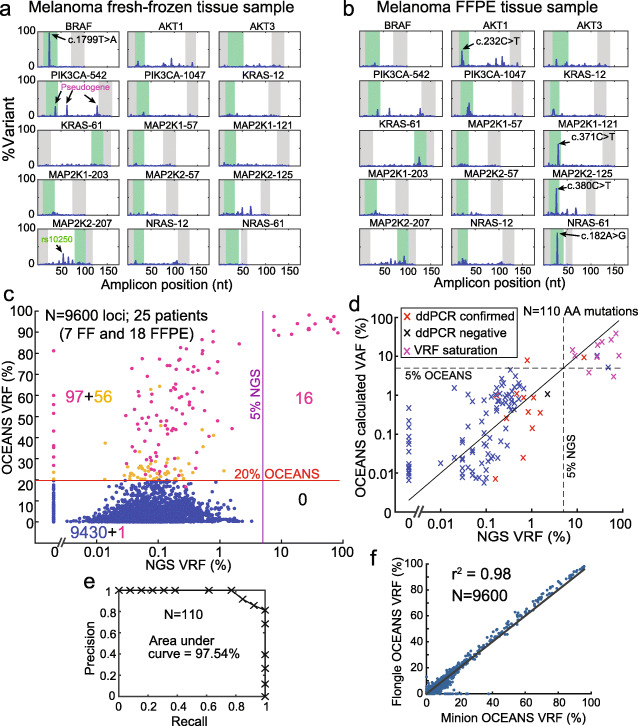
Validation of the 15-plex OCEANS melanoma panel on melanoma tissue samples. **a** Example results from a fresh-frozen melanoma tissue sample. The BRAF-V600E mutation was the only mutation called for this sample. **b** Example results from a formalin-fixed, paraffin-embedded (FFPE) tissue sample. 4 mutations were called in the AKT1, MAP2K1, MAP2K2, and NRAS genes. Other variants with ≥20% VRF that were not called by Clair are not labeled in the figure. All 4 of these mutations had confirmatory reads on a parallel NGS experiment, but only the NRAS c. 182A >G mutant had a NGS VRF of above 5%; see Additional file 1: Section S5 for details. **c** Summary of sequencing results for 25 clinical melanoma tissue samples (7 fresh/frozen, 18 FFPE). Input DNA quantities ranged from 10 ng and 50 ng (Additional file 2). The *X*-axis shows the VRF based on a standard NGS analysis, and the *Y*-axis shows the OCEANS VRF. The horizontal line shows the 20% VRF cutoff for OCEANS variant calls, and the vertical line shows the 5% VRF cutoff for NGS variant calls. The numbers in quadrants display the number of loci in each group; 97 of the 153 putative variants in the top-left quadrant also had a Clair score of above 180 (purple dots). Relative to the NGS results, the OCEANS panel had a sensitivity of 100% and a specificity of 99.0%, indicating a very a low false positivity rate. Importantly, we believe that many of the 97 NGS-negative and OCEANS-positive results were true mutations, and our ddPCR confirmation experiments support this hypothesis (Table 1). We did not observe any significant difference for fresh/frozen samples vs. FFPE samples (Additional file 1: Fig S12). **d** Comparison of OCEANS calculated VAF and NGS VRF. Original sample VAF was calculated for mutation calls from OCEANS VRF in panel (c) (Additional file 1: Section S4). Based on calibration experiments in Fig. 4 d, VAF quantitation dynamic range is relatively small due to VRF saturation. However, VAF estimation enables identification of high VAF mutations (>5%) to aid treatment decisions based on clinical diagnosis. Due to nanopore error rate, OCEANS %VRF >90% was considered as saturated and classified as high VAF mutation irrespective of the calculated VAF value. OCEANS identified all mutations with NGS VRF >5% as high VAF mutations. OCEANS called several low VAF mutations. Mutations confirmed by ddPCR are in red and a ddPCR negative mutation is indicated in black (Table 1). Note: 110 amino acid mutations (Additional file 2) from 114 mutated nucleotide loci that were called by Clair in panel (c) (pink dots) are plotted here. **e** Precision and recall for data in panel (d), based on changing the OCEANS calculated VAF cutoff. The area under the curve (AUC) is 97.54%. **f** High concordance of OCEANS results using Oxford Nanopore MinION vs. Flongle flow cells

**Validating OCEANS on clinical tissue samples.** We next applied the melanoma OCEANS panel to clinical melanoma tissue samples, including both fresh/frozen (FF) and FFPE tissue (Fig. 5 a, b). As in the calibration experiments, we called somatic mutations only when the VRF was observed to be greater than 20%, and the Clair score was above 180. In total, DNA from 7 FF and 18 FFPE tissue samples were sequenced using both OCEANS and NGS. The melanoma OCEANS panels cover a total of 384 loci, corresponding to a total of 9600 total loci analyzed across the 25 samples.

**Table 1:** Summary of OCEANS and ddPCR comparison experiments for 6 FFPE samples in 4 select mutation loci (BRAF p. V600E, KRAS p. G13D, KRAS p. E62K, and MAP2K1 p. P124L). Other than one sample/mutation combination at 31% VAF, ddPCR showed VAFs ranging between 0.02 and 0.66% for the concordant samples. See Additional file 1: Section S6 for detailed results

	OCEANS Pos.	OCEANS Neg.	Total
ddPCR Pos.	10	2	12
ddPCR Neg.	1	11	12
Total	11	13	24

Figure 5 c shows the comparison between OCEANS and NGS. All 16 somatic mutants called by NGS at above 5% VAF were also called by OCEANS, corresponding to a 100% OCEANS sensitivity relative to NGS. Of the 9584 NGS-negative loci, OCEANs called an additional 97 variants (Fig. 5 c); thus, relative to NGS, OCEANS had a 99.0% specificity.

We calculated the original sample VAF from OCEANS VRF using fold enrichment calculated for mutations in calibration experiments (Additional file 1: Section S5) [17]. The sequencing error rates combined with the saturation of VRFs near 100% after BDA enrichment means that our quantitation dynamic range is relatively small. However, estimation of sample VAF enables identification of high VAF (>5%) mutations to aid in making treatment decisions based on clinical diagnosis. OCEANS identified all mutations with NGS VRF >5% as high VAF mutations (Fig. 5 d). The OCEANS VRF and NGS VRF cannot be directly compared, since OCEANS VRF is not the true sample VAF. Therefore, precision-recall values were calculated from OCEANS calculated VAF and NGS VRF comparison in Fig. 5 d. By varying the OCEANS calculated VAF cutoff threshold, we can change the number of high VAF mutation calls that are verified by NGS as >5%, generating a set of precision/recall tradeoffs for detecting mutations with NGS VRF >5%, which can be plotted as a precision-recall curve (Fig. 5 e). Importantly, we believe that many of the 97 discordant called variants that were below the 5% NGS VRF cutoff could be real mutations, based on our calibration experiments. To confirm our discordant OCEANS mutation calls, we further performed digital PCR on 6 FFPE samples at 4 mutation loci (BRAF p. V600, KRAS p. G13D, KRAS p. E62K, and MAP2K1 p. P124L) and one fresh frozen sample for BRAF p. V600 loci (Supplementary excel table). Of these 25, 12 mutations were called positive by OCEANS and 13 were called negative by OCEANS. OCEANS was concordant with ddPCR for 11 positive samples and 11 negative samples (Table 1, Additional file 1: Section S6). Identification of such low VAF mutations makes OCEANS suitable for applications like Minimum Residual Disease (MRD) detection.

It is important to note that concordant positives between OCEANS and ddPCR indicate the existence of a DNA variant in the sample, which may not necessarily reflect a mutation in the patient. Cytosine deamination is a well-documented type of DNA damage frequently observed in DNA extracted from FFPE. We applied an FFPE damage repair kit to the FFPE DNA before performing OCEANS library preparation, but do not necessarily expect that all cytosine deaminations are repaired or excised. In particular, any repair kit based on cleaving/repairing uracils formed through the deamination of standard cytosine would not be able to rectify deamination of methylcytosines into thymines.

Because each ddPCR mutation requires a separate reaction, the ddPCR results required 4 times more input DNA than OCEANS just to cover these 4 mutations. For analysis of clinical biopsy samples, OCEANS would have significantly higher clinical sensitivity due to being able to analyze all mutations in the panel from a single sample.

Next, we wished to characterize the reproducibility and robustness of the OCEANS panel on different types of Nanopore Sequencing instruments and flow cells. The Oxford Nanopore Flongle flow cell, in particular, is relatively inexpensive at <DOLLAR/>70 and can further reduce turnaround time relative to MinION by reducing the need for sample batching before sequencing. We performed the OCEANS panel on all 25 melanoma samples on the Flongle, and observed highly quantitatively similar VRFs as our results on the MinION (Fig. 5 f).

**Fig. 6:**
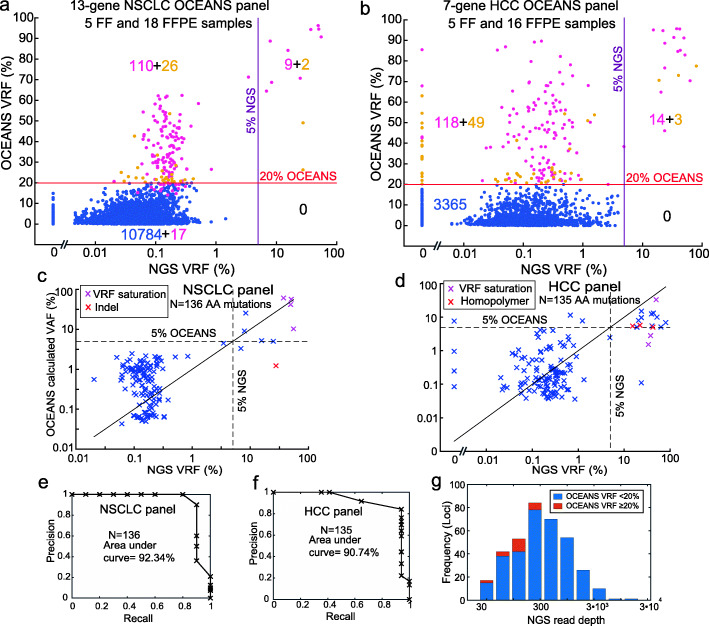
Validation of NSCLC and HCC OCEANS panels on clinical tumor tissue samples. **a** Summary of NSCLC panel results on 23 clinical samples (5 FF, 18 FFPE). Input DNA quantities ranged from 10 ng to 50 ng (Additional file 2). The *X*-axis shows the VRF based on a standard NGS analysis, and the *Y*-axis shows the OCEANS VRF. The horizontal line shows the 20% VRF cutoff for OCEANS variant calls, and the vertical line shows the 5% VRF cutoff for NGS variant calls. The numbers in quadrants display the number of loci in each group; 110 of the 136 putative variants in the top-left quadrant also had Clair scores of above 180 (purple dots). 2 of the 11 NGS confirmed variants in the top-right quadrant had Clair scores below 180 (yellow dots); both variants were insertions that typically have lower Clair scores. **b** Summary of HCC panel results on 21 clinical samples (5 FF, 16 FFPE). Input DNA quantities ranged from 10 ng to 50 ng (Additional file 2). 118 of the 167 putative variants in the top-left quadrant also had Clair scores of above 180 (purple dots). 3 of the 17 NGS confirmed variants in the top-right quadrant had Clair scores below 180 (yellow dots); all 3 variants were in TERT amplicon within a homopolymer region that resulted in lower Clair scores (Additional file 1: Section S5). **c, & d** Comparison of OCEANS calculated VAF and NGS VRF for NSCLC and HCC panel. Note: For NSCLC, 136 amino acid mutations (Additional file 2) from 136 clair called nucleotide changes in panel (a) (pink dots) are plotted here. For HCC, 135 amino acid mutations (Additional file 2)from 132 clair called nucleotide changes in panel (b) (pink dots) plus the 3 TERT mutations are plotted here. A insertion mutation in NSCLC and mutations in homopolymer regions in HCC are indicated red. VAF underestimation at these positions could be due to higher error rate. **e, & f** Precision-recall curve for data in panel (c) and (d), based on changing the OCEANS calculated VAF cutoff. **g** Panel (c) showed a significant number of loci wherein NGS results had 0% variant read frequency (VRF). Here, we show a histogram showing NGS read depth distribution for the 0% VRF loci, as well as the fraction of loci for each read depth group where OCEANS VRF was greater than 20%. Only loci with less than 300x NGS read depth showed discordance with OCEANS (mutation call based on 20% VRF)

**NSCLC and HCC OCEANS panels**. We next constructed two additional OCEANS panels: a 28-amplicon panel for non-small cell lung cancer (NSCLC) and an 11-amplicon panel for hepatocarcinoma (HCC) to show the generality of our approach. The NSCLC OCEANS panel covers roughly 1121 mutations in the COSMIC database across 13 genes (AKT1, ALK, BRAF, DDR2, EGFR, KRAS, NRAS, MAP2K1, MET, PIK3CA, PTEN, ROS1, and TP53, see Additional file 1: Section S4). DNA from 5 FF and 18 FFPE NSCLC tissue samples were sequenced using both OCEANS and NGS. Figure 6 a, c show the comparison between OCEANS and NGS. Nine out of 11 somatic mutants called by NGS at above 5% VAF were also called by OCEANS. The two mutations that had a Clair score less than 180 were indel mutations, for which Clair has been observed to be less accurate [10, 25].

The HCC OCEANS panel covers roughly 680 mutations across 7 genes (CTNNB1, ARID1A, AXIN, TERT, JAK1, PTEN, and TP53, see Additional file 1: Section S4). DNA from 5 FF and 16 FFPE HCC tissue samples were sequenced using both OCEANS and NGS. Figure 6 b, d show the comparison between OCEANS and NGS. Fourteen out of 17 somatic mutants called by NGS at above 5% VAF were also called by OCEANS. The 3 mutations not called by Clair were in the TERT amplicon within a homopolymer region (Additional filer~efMOESM1: Section S5). Higher Nanopore Sequencing error rates in homopolymer regions could be the reason for lower Clair scores despite the OCEANS VRF being >70% for these mutations. We observed 23 loci with OCEANS VRF greater than or equal to 20% for which the corresponding NGS VRF were 0%. We analyzed the NGS read depth for all loci with NGS VRF equal to 0% and their corresponding OCEANS VRF (Fig. 6 g). All 23 loci with OCEANS VRF greater than or equal to 20% had NGS read depth of less than 300. Overall, both OCEANS panels had high concordance between OCEANS and NGS. The area under the precision-recall curve was 92.34% for the NSCLC panel and 90.74% for the HCC panel based on OCEANS calculate VAF (Fig. 6 e, f).

## Discussion

The rapid turnaround time and low instrument/consumables cost of Nanopore Sequencing render Nanopore Sequencing attractive for time-sensitive therapy selection and recurrence monitoring applications in oncology. Although multi-nucleotide indels, copy number variations, and gene fusions can be detected at low VAFs by Nanopore Sequencing, previously the rapid detection of single nucleotide somatic mutations with low VAFs has not been demonstrated on the Nanopore Sequencing platform. The OCEANS method we present detects single-base mutations from FFPE tissue-derived DNA with LoD of less than 1% with a 10-h workflow and positions Nanopore Sequencing to transition into clinical sequencing panels (Table 2). Within OCEANS, BDA enhances the confidence and sensitivity of somatic mutation calls, and SAL reduces the turnaround time by enhancing the throughput of Nanopore Sequencing. However, the latest and upcoming improvements to ONT’s sequencing chemistry and basecalling algorithms [36] may remove the need for SAL to get higher throughput with short DNA libraries, enabling BDA to be applied directly for Nanopore Sequencing.

**Table 2:** Comparison of sequencing methods

	OCEANS	Standard NGS	NGS w/ UMI
	(MinION)	(MiSeq)	(MiSeq)
Samples / flow cell	24	24	2
Flow cell price	<DOLLAR/>900	<DOLLAR/>1300	<DOLLAR/>1300
Flow cell reusability	10	1	1
Library preparation kits price	<DOLLAR/>890	<DOLLAR/>750	<DOLLAR/>63
Sequencing price / sample	<DOLLAR/>7.45	<DOLLAR/>85.4	<DOLLAR/>682
Turnaround time	10 h	24-72 h	24–72 h
Single-base VAF LoD	0.05–1%	2–5%	0.1–0.5%

For liquid tumors and for solid tumors where fresh/frozen tissue biopsy samples are available, the long read length of Nanopore Sequencing further enables accurate detection of large-scale DNA structural variations. Integrating OCEANS with existing Nanopore Sequencing experimental and bioinformatics methods for structural variant profiling would allow for the development of comprehensive oncology panels covering a broad range of structural alterations observed in leukemias and lymphomas. In contrast, the DNA from FFPE tissue samples and cell-free DNA from plasma are short and do not physically allow for direct long-read sequencing to identify large-scale alterations. We used Clair variant caller that is based on DeepVariant variant calling methodology, since it has been shown to have the highest accuracy for calling mutations on Nanopore Sequencing data [21, 32]. Improvements in variant calling software for Nanopore Sequencing will improve the specificity and sensitivity of OCEANS especially for in-del mutations. Here, we used SQK-LSK109 sample prep chemistry on R9.4.1 flow cells and MinKNOW 19.12.5 (fast basecalling model) for basecalling. The latest R10.3 flow cells in combination with ONT’s upcoming Q20+ sequencing chemistry with improved accuracy and throughput may further enhance OCEANS performance and turnaround time for low VAF detection.

In this work, our bioinformatics pipelines make qualitative variant calls based on a combination of observed VRF and Clair score. We used the fold-enrichment calculated for each BDA design and inferred the sample VAF from VRF for OCEANS (Additional file 1: Section S4) [17]. This enabled us to identify high VAF (≥5%) and low VAF mutations. However, the sequencing error rates combined with the saturation of VRFs after BDA enrichment mean that our quantitation dynamic range is relatively small. We underestimated the sample VAF if the VRF saturates or if the sequencing error rate at that particular mutation position was high. We overestimated the sample VAF for mutations like deletions and insertions of multiple nucleotides that resulted in a higher enrichment fold than that of the single nucleotide mutations used in calibration experiments. Integration of OCEANS with unique molecular identifier (UMI) barcodes [23, 24] will likely be necessary for accurate VAF quantitation on the Nanopore Sequencing platform.

## Conclusions

We developed a method (OCEANS) to selectively amplify variants with low VAFs and subsequently concatenate the amplicons for Nanopore Sequencing. OCEANS allows accurate detection of somatic mutations with VAF limits of detection between 0.05 and 1% by Nanopore Sequencing. On clinical FFPE tumor samples OCEANS showed 99.79 to 99.99% area under the receiver-operator curve in comparison experiments against Illumina NGS. Comparison against digital PCR on 11 putative mutations at ≤1% VAF showed 10 concordant positive calls with VAFs between 0.02 and 0.66%. The rapid turnaround time and low instrument/consumables cost of Nanopore Sequencing is designed to enable same-day clinical sequencing panels. Integrating OCEANS with long-read and base modification detection capabilities of Nanopore Sequencing can enable development of comprehensive oncology panels.

## Methods

**Oligonucleotides.** Primers and blockers (Sequences are provided in Additional file 3) were purchased from Integrated DNA Technologies, as well as gBlocks that serve as positive controls. All DNA oligos were purchased standard desalted and dissolved in 1x TE buffer (10 mM Tris-HCl, 1 mM EDTA, pH 8). Dilutions of gBlock DNA were done in 1x TE buffer with 0.2% Tween 20 (Sigma) and 100 ng/ *μ*l Carrier RNA(poly A)(Qiagen, Catalog No.1017647).

**Repository DNA samples.** Human gDNA samples (NA18537 and NA18562) were purchased from Coriell Biorepository. BRAF V600E reference standard (HD238) and Myeloid DNA reference standard (HD829) were purchased from Horizon Discovery. Seven melanoma fresh frozen patient tissue samples were purchased from OriGene. Twenty-one melanoma FFPE patient tissue samples were obtained deidentified from MD Anderson Cancer Center. Five HCC and five NSCLC fresh frozen patient tissue samples were purchased from OriGene. Fourteen HCC FFPE patient tissue samples were purchased from US Biolab and two HCC FFPE patient tissue samples were purchased from OriGene. Eighteen NSCLC FFPE patient tissue samples were purchased from OriGene. DNA from fresh frozen samples were extracted using QIAamp DNA Mini Kit (Catalog No. 51304). DNA from FFPE samples were extracted using Qiagen GeneRead DNA FFPE kit (Catalog No.180134) and repaired using NEBNext FFPE DNA repair mix (NEB #M6630L).

**Stochastic Amplicon Ligation (SAL).** BsaI restriction sites and complementary overhangs for assembly were appended to DNA by PCR using SAL adapter primers and Phusion Hot Start Flex DNA polymerase (NEB M0535S). For single-plex reactions, the following thermocycling protocol was used: 98 ^∘^C, 30s (98 ^∘^C, 20s; 63 ^∘^C, 30s; 72 ^∘^C, 30s) × 3 (98 ^∘^C, 20s; 72 ^∘^C, 1 min) ×*n*; 72 ^∘^C, 5 min. For multiplex panels, the following thermocycling protocol was used: 98 ^∘^C, 30s (98 ^∘^C, 20s; 63 ^∘^C, 2 min; 72 ^∘^C, 2 min) × 3 (98 ^∘^C, 20s; 72 ^∘^C, 3 min) ×*n*; 72 ^∘^C, 5 min. The number of PCR cycles (*n*) was empirically determined based on the amount and nature of input DNA. Amplicons from PCR were purified by column purification with Monarch PCR & DNA Cleanup Kit (NEB #T1030). DNA was quantitated by Invitrogen Qubit dsDNA HS (High Sensitivity) Assay Kit (Catalog number: Q32851). NEB BsaI-HFv2 kit (E1601S) was used for amplicon assembly. A 20 *μ*l reaction was set up containing 400–500 ng of DNA and 2 *μ*l golden gate enzyme mix in 1x T4 DNA ligase reaction buffer. Reaction was cycled for 30 times, with each cycle containing an incubation at 37 ^∘^C for 2 min and at 16 ^∘^C for 2 min. The assembled DNA was size selected twice using 0.4x Agencourt AMPure XP beads (Beckman Coulter A63881).

**Oncogene Concatenated Enriched Amplicon Nanopore Sequencing (OCEANS).** Ten to 50 ng of human genomic DNA sample was mixed with primers and blockers and subject to PCR using Phusion Hot Start Flex DNA polymerase. For single-plex tests, 400 nM of the forward and reverse primer and 4 *μ*M blocker were used. For the 7-plex AML panel, 75 nM of each forward and reverse primer and 750 nM of each blocker were used. For the 15-plex melanoma panel and 11-plex HCC panel, 50 nM of each forward and reverse primer and 500 nM of each blocker were used. For the 28-plex NSCLC panel, 15 nM of each forward and reverse primer and 150 nM of each blocker were used. For single-plex, the following thermocycling protocol was used: 98 ^∘^C, 30s (98 ^∘^C, 10s; 63 ^∘^C, 30s; 72 ^∘^C, 30s) × 23; 72 ^∘^C, 5 min. For AML and Melanoma multi-plex panels, the following thermocycling protocol was used: 98 ^∘^C, 30s (98 ^∘^C, 20s; 63 ^∘^C, 2 min; 72 ^∘^C, 2 min) × 23; 72 ^∘^C, 5 min. For HCC and NSCLC multi-plex panels, the following thermocycling protocol was used: 98 ^∘^C, 30s (98 ^∘^C, 10s; 63 ^∘^C, 5 min; 72 ^∘^C, 30s) × 23; 72 ^∘^C, 5 min.

The amplicons were then purified by column purification and used as input for PCR using SAL adapter primers and subject to SAL as described above. Assembled DNA was used for library preparation using Ligation sequencing kit (SQK-LSK109) following the protocol provided by ONT. Briefly, 50–200 ng of assembled DNA was end-repaired and dA tailed using NEBNext Ultra ^*TM*^ II End Repair/dA-Tailing Module (E7546) and purified using 1x AMPure XP beads. Native barcoding kit (EXP-NBD104 or EXP-NBD114) was used for barcoding. Forty to 100 fmol pooled barcoded DNA was used for adapter ligation. The ligation reaction was purified using 0.5x AMPure XP beads. The beads were washed using S Fragment Buffer (SFB) and eluted in Elution Buffer (EB). The eluate was quantified by Qubit and then loaded on to Minion R9.4.1 flow cells and sequenced for 40 min. For Flongle flow cells, 20–50 fmol pooled barcoded DNA was used for adapter ligation, and sequencing was run for 8–10 h.

**Next-generation sequencing (NGS) verification of clinical samples on Illumina.** Ten to 50 ng of genomic DNA extracted from FFPE samples was used as input, for multiplex PCR amplicon sequencing. The following thermocycling protocol was used: 98 ^∘^C, 30s (98 ^∘^C, 20s; 63 ^∘^C, 2 min; 72 ^∘^C, 2 min) × 15; 72 ^∘^C, 5 min. The amplicons were purified by column purification. NEBNext ultra II DNA library prep kit for Illumina (NEB # E7645S) was used for library preparation following the kit protocol. NEBNext Multiplex Oligos for Illumina (Dual Index Primers Set 1) (NEB #E7600S) were used for index PCR. Sequencing was done on Illumina MiSeq using the V2 kit. Each sample was sequenced to at least 10,000x coverage. Libraries were checked by running on Bioanalyzer before sequencing.

**ddPCR quantitation protocol.** ddPCR assays were performed on QX200 Droplet Digital PCR system (Bio-Rad) using BRAF V600E (Bio-Rad dHsaMDV2010027), BRAF V600K (Bio-Rad dHsaMDV2010035), KRAS p.E62K (Bio-Rad dHsaMDS453364969), KRAS p.G13D c.38G >A (Bio-Rad dHsaMDV2510598), and MAP2K1 p.P124L (Bio-Rad dHsaMDS897199134) mutation detection kits according to kit protocols.

**Nanopore Sequencing Bioinformatic analysis**. Nanopore Sequencing reads were basecalled using MinKNOW 19.12.5 (fast basecalling model). Barcoded Nanopore Sequencing fastq reads were demultiplexed using EPI2ME software (Oxford Nanopore Technologies). Typically, 10,000 Nanopore Sequencing reads were used per sample for analysis. The SAL reads were deconcatenated using a custom python script [33] and the deconcatenated reads were aligned to human reference genome (GRCh38) using minimap2 aligner (https://doi.org/https://github.com/lh3/minimap2) to generate a bam file. IGVtools (https://doi.org/http://software.broadinstitute.org/software/igv/download) was used to extract the number of A, C, G, & T basecalled nucleotides, insertions, and deletions at each position of the amplicon using the basecount command in IGV-command-line tools. The VRF at each nucleotide position was calculated as the highest frequency single-base change at that position.

Bioinformatic workflow for variant calling using Clair variant caller is summarized in Figure S9. Clair v1 (https://doi.org/https://github.com/aquaskyline/clair) was used for variant calling. First, the bam file generated using minimap2 was down-sampled to <150x coverage for each amplicon using samtools view command (Subsampled read names are provided in Additional file 5). The subsampling was necessary because Clair was trained on whole genome sequencing data with low coverage. Therefore, using high coverage results in a higher false positivity rate [21]. The subsampled bam file was then used to call variants using callVarBam submodule and ont r94-flipflop model. Allele frequency threshold of 0.2 and minimum coverage of 50x were used. Variant calls were filtered for score >180.

**NGS Bioinformatic analysis**. NGS reads were trimmed of adapter sequences and aligned to reference amplicon sequence using short read (sr) function of minimap2 aligner. IGVtools (http://software.broadinstitute.org/software/igv/download) was used to extract the number of A, C, G, & T basecalled nucleotides, insertions, and deletions at each position of the amplicon using the basecount command in IGV-command-line tools [31]. The VRF at each nucleotide position in the enrichment region (nucleotide positions covered by the blocker in BDA) was calculated as the highest frequency single-base change at that position. Mutations in the enrichment region with VRF >5*%* and minimum coverage of 80x were considered true positives.

## Supplementary Information

**Additional file 1** Supplementary information

**Additional file 2** Mutation summary - Summary of Nanopore and NGS mutation calls in clinical samples

**Additional file 3** OCEANS oligo list - Oligo sequences used

**Additional file 4** SRA accessions - Detailed list of SRA accessions

**Additional file 5** Subsamples read names - Subsampled read names used for variant calling with Clair

**Additional file 6** Review history

## Data Availability

Custom bioinformatics software was written in python for Nanopore Sequencing reads processing and is available here: https://doi.org/https://github.com/aqueous87/snippyunder opensource license Mozilla Public License 2.0 and from Zenodo [33]. Nanopore and NGS sequencing data was deposited in NCBI Sequence Read Archive (SRA) under Bioproject accession PRJNA727742 [34]. Detailed list of SRA accessions is available in Additional file 4.
