# Supplementary material for: Oncogene Concatenated Enriched Amplicon Nanopore Sequencing for rapid, accurate, and affordable somatic mutation detection
Source: Genome Biol. 2021 Sep 6;22:227. doi: 10.1186/s13059-021-02449-1 (PMC8419911; doi:10.1186/s13059-021-02449-1)
Supplement: Supplementary file 1 — Additional file 1 Supplementary information [file 13059_2021_2449_MOESM1_ESM.pdf]

Oncogene Concatenated Enriched Amplicon Nanopore Sequencing for Rapid, Accurate, and Affordable Somatic Mutation Detection Supplementary Information

Deepak Thirunavukarasu, Lauren Y. Cheng, Ping Song, Sherry X. Chen, Mitesh J. Borad, Lawrence Kwong, Phillip James, Daniel J. Turner, and David Yu Zhang

(Dated: May 1, 2021)

S1. Effect of DNA Length on NS Throughput 1
S2. SAL Design and Performance 3
S3. Bioinformatic Analysis 6
S4. Analytical Validation Experiments with Synthetic DNA 10
S5. Mutations Detected in Melanoma Clinical Samples and NGS Comparison 15
S6. ddPCR Comparison 18

Section S1. Effect of DNA Length on NS Throughput

NS of 160 bp vs 10 kb fragmented human genomic DNA. Fig. S1 shows gel images of human genomic DNA fragmented to mean length of 160 bp or 10 kb. NS read statistics of the two libraries are shown in Fig. S2 and Fig. S3. The mean read length of 160 bp fragmented library was 265 bp, due to the attachment of barcodes and sequencing adapter prior to NS. The 10kb DNA library had a mean read length of 3.8 kb. The Q-score of 10 kb library was higher than that of the 265 bp library.

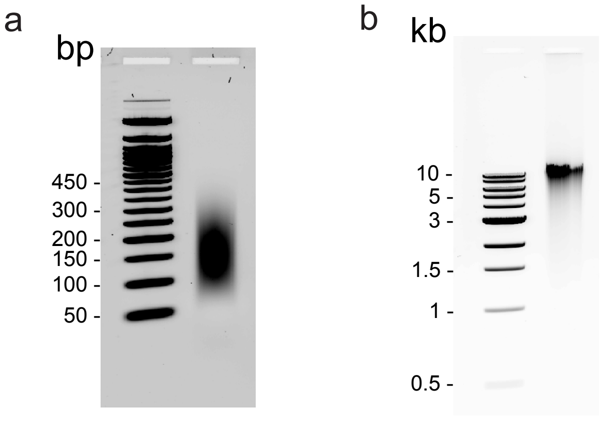

FIG. S1: Size verification of fragmented human genomic DNA. **(a)** 160 bp library run on a 2% agarose gel. **(b)** 10 kb library run on a 0.5% agarose gel.

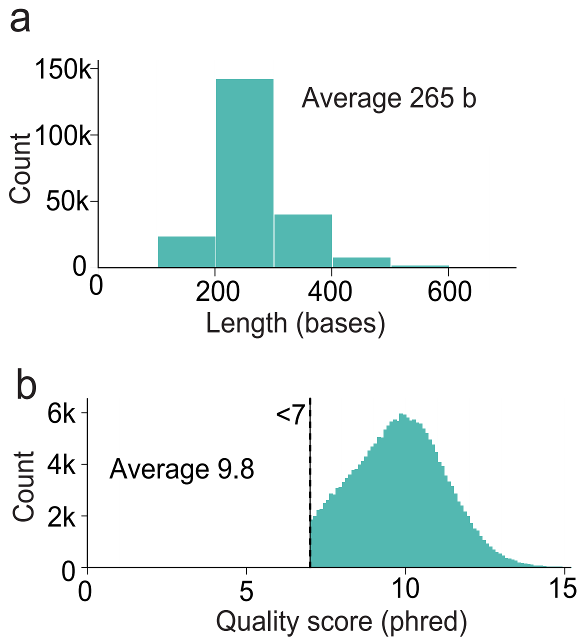

FIG. S2: NS run performance of 160 bp fragmented library. **(a)** Length distribution of NS reads. **(b)** Q-score distribution of NS reads.

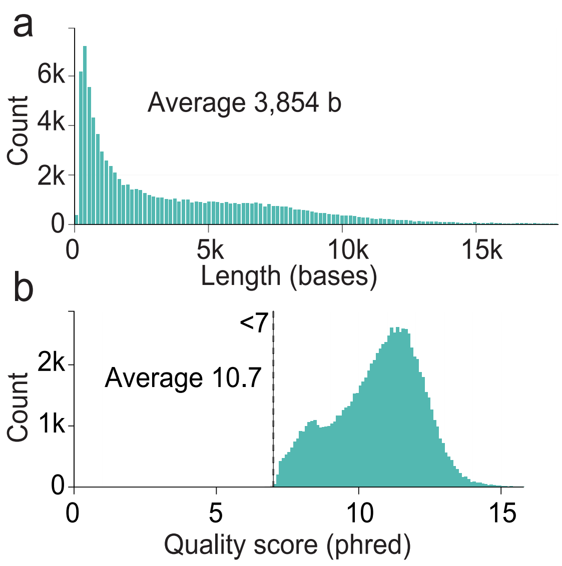

FIG. S3: NS run performance of 10 kb fragmented library. **(a)** Length distribution of NS reads. **(b)** Q-score distribution of NS reads.

Section S2: SAL Design and Performance

Fig. S4 shows the design of SAL adapter sequences. Fig. S5 shows agarose gel electrophoresis analysis of SAL concatemer products before and after exonuclease digestion. The remaining bands after digestion are likely circular concatemer products. NS throughput for original amplicons vs. SAL products are shown in Fig. S6. Fig. S7 shows the individual NS amplicon traces for 0% and 5% VAF samples shown in Fig. 2e.

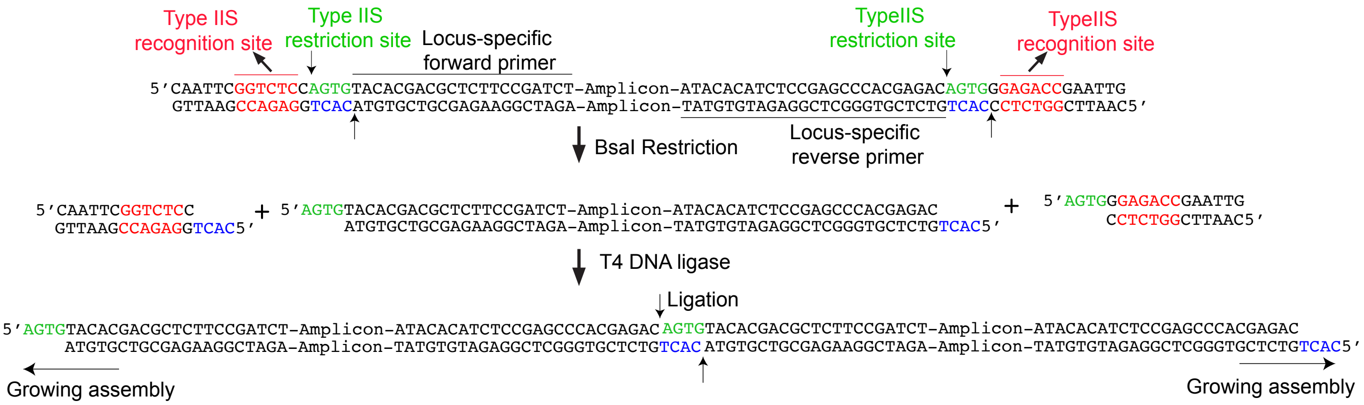

FIG. S4. Schematic of SAL design. Adapter sequences containing type IIS recognition sequence (red) followed by restriction sites are attached to the ends of amplicons by PCR. After cleave by the type IIS restriction enzyme BsaI, complementary 4 base overhangs (green and blue) are generated. During the ligation step, two amplicons hybridize through their complementary ends are ligated. The DNA assembly grows at both ends by cycling between restriction and ligation steps.

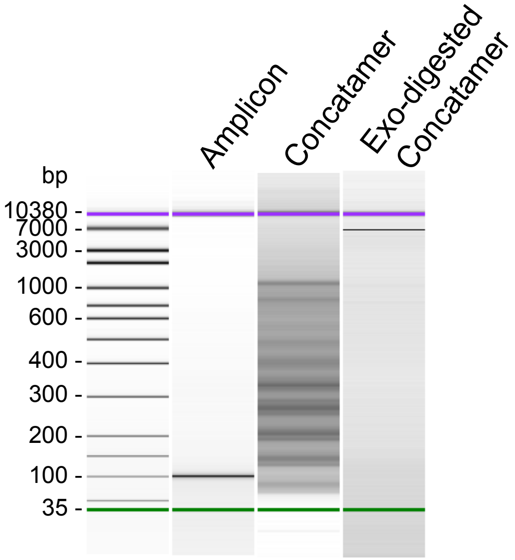

FIG. S5. Exonuclease treatment of SAL concatemers. 25 ng of SAL assembled concatemers of 100 bp DNA were treated with 10U of T7 exonuclease and 10U of Exonuclease VII to digest linear dsDNA.

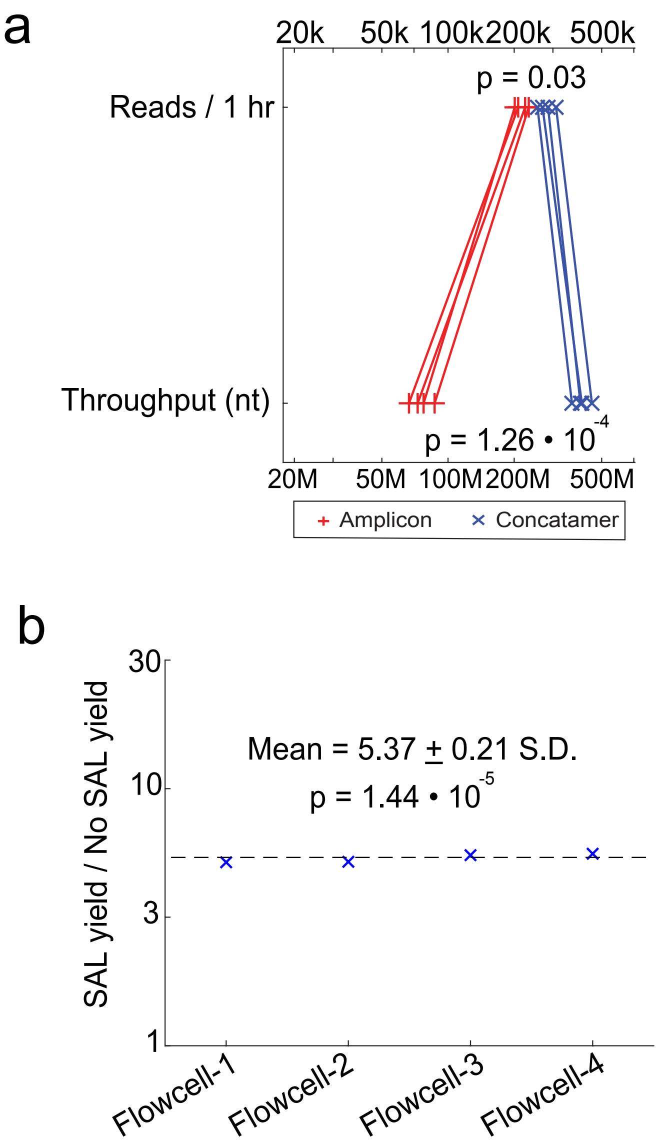

FIG. S6. NS throughput when sequencing amplicons vs. SAL concatemers. **(a)** NS reads and throughput for amplicons vs. concatamers. Here, PCR amplicons of size 180bp were assembled by SAL. The amplicon without SAL and after SAL were sequenced on four new flow cells for 1 hour each. In two flow cells, the amplicon library was sequenced first, the flow cell was then washed and loaded with the SAL library. The order of loading was reversed for the other two flow cells. p-value was calculated using two-way Student's t-test. **(b)** Ratio of yield of direct amplicon library to SAL library on each flow cell plotted on log scale, with p value calculated via a one-way Student's t-test.

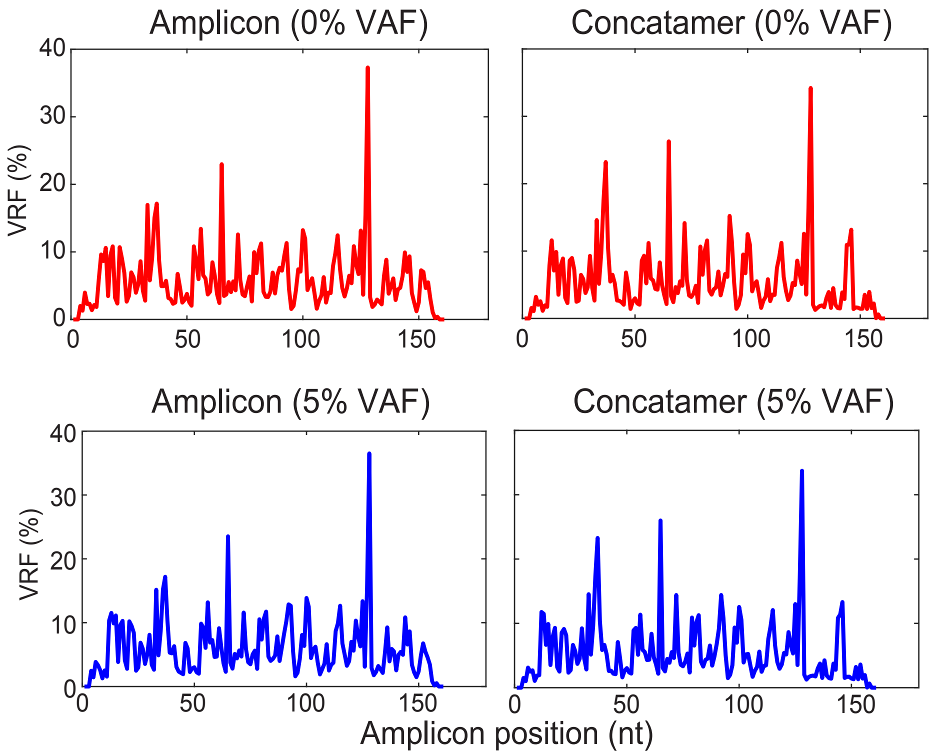

FIG. S7. NS amplicon traces with and without SAL. Top panels show amplicon traces for 0% VAF sample. Bottom panels show amplicon traces for 5% VAF sample. VRF of NS reads at each location that corresponds to the highest frequency single-base changes at that position. ΔVRF in Fig. 2e was calculated by subtracting VRF of 0% VAF sample from 5% VAF sample.

Section S3: Bioinformatic Analysis

Summary of bioinformatic workflow. NS on MinION was run for 30 min to 1 hour depending on the number of barcoded samples. Typically, 350k-500k reads were obtained in the first one hour of NS. Base-called reads were demultiplexed using EPI2ME software (Oxford Nanopore Technologies). Depending on the total number of reads obtained per sample, reads were subsampled randomly to approximately 10,000 reads per sample for analysis. The NS reads were then deconcatenated using a custom python script.

The python script used minimap2 (github.com/lh3/minimap2) to map individual wildtype amplicon reference sequences to the concatenated reads and extract the mapped monomer sequences from the concatenated read. This step also removed any off-target amplicon sequences from the concatenated reads. Fig. S8 shows a sample concatenated read.

The mapping efficiency for finding monomer sequences within concatenated reads was calculated as mapped nucleotide fraction (MNF), the fraction of concatemer reads that mapped to an amplicon of interest (Fig. S8). Manual analysis of concatenated reads showed that minimap2 missed some monomers (Fig. S8). Tables S1 and S2 shows the number of reads analyzed for each sample and the MNF values. MNF was higher for FFPE samples, which could be due to the fragmented nature of DNA that resulted in reduced amplification of off-target sequences. Sub-sampling different sets of approximately 10,000 reads resulted in same variant calls with similar % VRF values (Table S3).

The mapped on-target sequences obtained after deconcatenation were then analyzed in two ways for variant calling. First, the sequences were aligned to amplicon reference sequences using minimap2 aligner to generate a BAM alignment file. IGVtools was used on the BAM file to extract the number of A, C, G, and basecalled nucleotides, insertions and deletions at each position using the basecount command. The VRF at each nucleotide position was calculated as the highest frequency single-base change at that position. Positions with VRF ¿20% were flagged as potential variants.

Second, the sequences were aligned to human reference genome (GRCh38) using minimap2 aligner to generate a BAM alignment file. The BAM file was down-sampled to <150x coverage for each amplicon using samtools view command. The downsampled BAM file was then used with Clair to call variants. Variant calls with score > 180 were flagged as potential variants. The bioinformatic workflow for variant calling using Clair variant caller is summarized in Fig. S9. Potential variants with both VRF> 20% and CLAIR score > 180 were definitively called as variants.

Sample SAL concatenated read (2925 nt)

CAATTCGGTCTCCCACTAGATGATGGGCTCCCGGAAGACAGTCCCCCAGGATGTTCGGATAGTTCCATTGGGACTTTCCACATCTTCT CACTCATCTGCAAAAACATCCCACGCCCC
TAGTCCCTGGCTGGACCAAGCCCATCACCATTGGCAGGCACGCCCATGGCGACCAACACTGTGTTGAGATGGACCCCCTATTTATGGATTTATTTGTATTTGCCTTTAGCTAAATGTG
TGTAAATACAGTTATACATATATGCATTCTCAATTTCATACCTTGCTTAATGAGGTGTAGATACCCAAAGATAAAGAATAAAAACACATACAAGTTGGAAATTTCTGGGCCATGAAAAA
AAAACATGCAAATCACATTATTGCCAACATGACTTGCCAGTCCCATAAGCATGACAACCTATGATGATAGGTTTACCCATCCTCAAAAGCCACTCATCTGCAAAAACATCCCACGCCT
AGTCTAAGCTGCGTTGGCCCCATCACCGGTGGCAGGCACGCCCATGGCGACCAGCACTAGATGATGGGCTCCCGAAGTAGTCCCCCAGGGGATGTTGAATGGTTCCATTGGGACT
TTCACATCTTCTCACTAGTTAGTTTTCACTACTACAAGTTAAAATGAATTTAAATAGTTTCTTTCTCCTCCAACTAATAGTGTATTCCACAGAGACAGCAGCCAGAAATATCCTCCTTAC
TCATGGTCGGATCACAAGATTTGTGATTTTGGTCTATCAGACACATCAAGAATGATTCTAATTATGTGGATTTAAAGGACACTGAATGGGCTCCCAGAAGACAGTCCCCCAGGATGTT
CCAGATAGTTCCGTGGGACTTTTCCACATCTTCTCACTAGATGATGGGCTCCCGGAAGACAGTCCCCCAGGATGTTCAGATAGTTCCATTGGGACTTTTCCACATCTTCTCACTTGTG
TTGAGATGGACAACCTATTTGTAAAGTTTATTTGTATTTGCCTTTAGCTAAATGTGTGTAAATATATACAGTTATACATATGCATTTCTCAATTTCATACCTTGCTTAATAGATTGTAGATAC
CAAAGATAAGAATAAAACACATACAAGTTGGAAATTTCTGGGCCATAGAAAAAAAAAAAAAAACATGCAAAATCACATTATTGCCAACATGACTTGCTTGATCCCCATAAGCATGACG
ACCTATGATGATAGGTTTTACCCATCACTCACAAGCCACTAGATGGTGGGCTCCCGGTGGCATTTCTTGCCCGACCTGAGGATGTACCCGCCACGCCTGCAGGACTGACCTCTAGGT
GGGCAAGCCGAGGCACAAGAGGGCGCGCTCTGGCGGAGTCAGCCCTCTTGTGAATTTGGGCTGGGAGACCAGCCAGGCCTAACAGGCAAGGCTGGGTCAGGCGAGGTCCTGG
AGCCCACTTCGGTGTAGAGTGTGCAAAATGTGAACAGGCCTGCCCAGGATAGCTCCTGCATTAGCGCTGGTGCCTTACGAGCGGCCAGTCAGATTTTTATTTGGCACCACTACAGA
GAGACCCAGGAGAGTCTCTTTAAGAAAATAGTTTAAACCACTAGATGATGGGCTCCCGGAAGACAGTCCCCCAGGATGTTCGGATAGTTCCGTGGGACTTTTCCATCTTCTCACTCC
CGCCAGGAACGTGCTTGTCACCCACGGGAAAGTGGTGAGAATATGTGACTTTGGATTAGCTCGAGTTATCATGAGTGATTCCAACTATGTTGTCAGGGCACTCATCTGCAAAAACAT
CCACACTAGTCCCTAGCTGGACCAAGCCCATCACCATTGGCAGGCACGCCCATGGCGACCAGCACTAGATGGGCTCCGGAAGACTGATCCCCAGGATGTTCCGGATAGTTCCATTG
GGACTTTCCACATCTTCTCACTCCGCCAAGGAACGTGCCTTGTCACCCACGGGAAAGTGGTGAAGATATGTGACTTTGGTTGGCTCGAGTTATCATGAGTGATTCCAACTATGTTGT
CAGGGCACTAGATGATGGGCTCCCCGGAAGACAGTCCCCAGGATGTTCCAGATAGTTCCATTGGGACTTTTCATCTTCTCACTAGATGATGGGCTCCCGGAAGACAGTCCCCCAGG
ATGTTCCAGATAGTTCCATTGGGACTTTTCATCTTCTCACTAGAAGATTTCCTTGGAACTAAGCAGGCGTCAGAGGAGTTGGTGGGTGTGAGTGCCCTGTCCCTGCACTTCGGGTGG
CTGCTGGTCCTCAGGTCCTGCTGTGTGGTTAGACGGCTTCCGGGCAGCCTGGTCTGGCCAACACTCACCTACCCTCTCTGCCTTTTCTCCCCAGAATTGTTGGTTTCCCAGTCCACTA
TACTGACGTCTCCAACATGAGCCGCTTGGCGAGGCAGAGACTGCTCACTTTAAAGTGTTTGGAATTAAATTACATCTAATTATAAAAATTTTCTTGGAGTCATATCTTTATCTAGAGTTA
ACTCTCTGAGTGGTAGAATGAAAAAAACAGATGTTGAACTATGCAAGAGACATTTGAATTTATTGATGTCTATGAAGTGTTGTGGTTCCTTAGCCACATTTCTTTTTTTCAGGCTATTC
AAGATCTCTGCATGGCAGTGGAGGAAGTCTCTTTAAAATAGTTTAAACCACTTTAAAGTGTTTGGACTAAATTACAAATACGAGTATAGGGTACTGCTTGGAGTCATATCTTTATCTAG
AGTTAACTCTCTGGTGGTAGAATGAAAGTAGATATTGAACTATGCAAAGAGACATTTAATTTGATGTCTGTGAGTGTCATTCTGTTCATCTCCATCATGGCGGTGGAGGAAGTCTCTTT
AAGAAAATAGTTTAAACCACTAGATGATGGGCTCCCGGCCTCCTCTCCATTCAATGCCTGCCCCAACTCCCTGAGCTCTAGCTCCGCTGGTCTCCTCCGAGG

Amplicon monomers: DNMT3A KIT NPM1 IDH1 IDH2-140 IDH2-172 FLT3 Off-target

$$\textbf{Mapped nucleotide fraction (MNF)} = \frac{\text{[Length of Mapped (Underlined) reads]}}{\text{[Length of concatamer]}} \times 100\%$$

$$= \frac{1711\ \text{nt}}{2925\ \text{nt}} \times 100\% = 58.5\%$$

FIG. S8. Sample SAL concatenated read obtained on the OCEANS AML panel. Different colors show the 7 different amplicon sequences of the AML 7-plex panel. All the amplicons of the panel happen to be present in this concatenated read. Two off-target (brown) sequences are also present. CACT sequence (black) is the 4 nucleotide overhang sequence used in the SAL design. Underlined sequences are those that were mapped by minimap2 and deconcatenated by the custom python code. The deconcatenated MNF was calculated to be 58.5%.

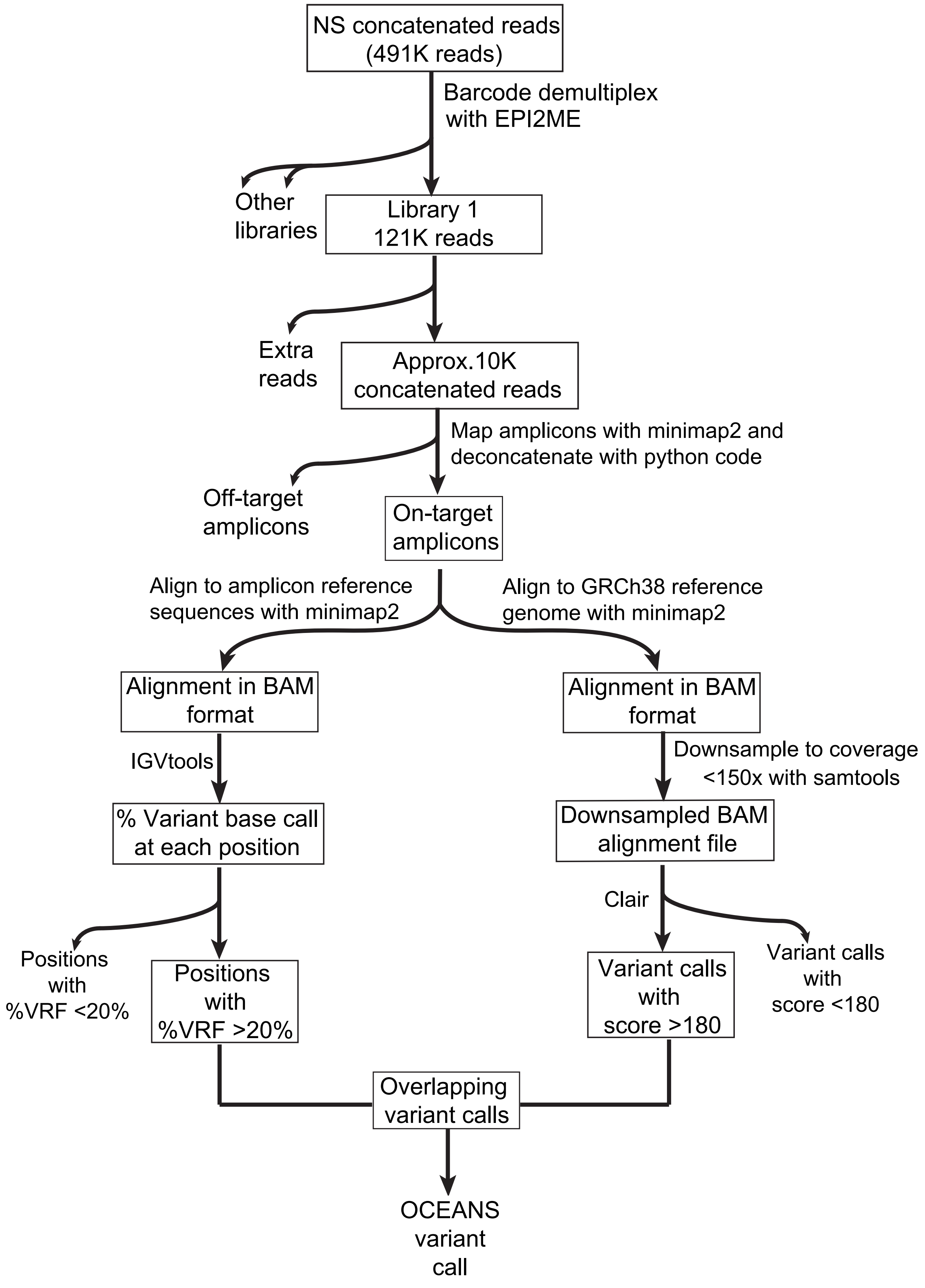

FIG. S9. Schematic of bioinformatic workflow for OCEANS variant calling.

Sample	NS reads	NS throughput (Mb)	Down-sampled reads	MNF
Synthetic 0%	40371	38.84	9948	22.66%
Synthetic 0.05%	47480	40.24	10548	32.07%
Synthetic 0.1%	46662	39.36	10361	35.05%
Synthetic 0.2%	48400	39.20	10916	41.17%
Synthetic 0.5%	49076	40.81	10920	45.14%
Synthetic 1.0%	38956	36.74	9777	48.71%
Horizon HD238	103621	89.98	11366	33.38%
Clinical FF2	106152	102.89	8305	33.25%
Clinical FF3	100151	103.39	8321	29.37%
Clinical FF20	101103	95.33	8601	41.76%
Clinical FF26	99803	91.88	7951	38.05%
Clinical FF52	110598	103.63	8762	50.48%
Clinical FF61	121686	106.37	9049	29.53%
Clinical FF172	134055	104.32	10165	38.43%
Clinical FFPE3	167541	119.61	8965	54.34%
Clinical FFPE4	131576	104.35	9066	61.42%
Clinical FFPE5	124667	104.31	11127	63.51%
Clinical FFPE7	152421	115.09	9986	60.51%
Clinical FFPE8	127153	96.82	9429	59.97%
Clinical FFPE10	115243	87.26	10381	62.78%
Clinical FFPE12	141370	110.07	10879	57.78%
Clinical FFPE13	130721	112.89	9565	51.72%
Clinical FFPE14	157380	119.88	9137	53.53%
Clinical FFPE15	118950	105.75	9055	60.53%
Clinical FFPE17	179188	114.15	9999	49.36%
Clinical FFPE18	123029	92.40	10013	65.05%
Clinical FFPE19	160886	120.33	10643	58.53%
Clinical FFPE20	178399	133.02	9844	58.3%
Clinical FFPE21	135317	108.29	10147	58.42%
Clinical FFPE23	118125	99.96	8095	54.16%
Clinical FFPE24	118941	97.34	9592	56.26%
Clinical FFPE25	120325	108.32	9886	54.7%

TABLE S1. Number of reads obtained and sampled for OCEANS melanoma panel, and the MNF for each sample.

	DNMT3A	IDH1	FLT3	IDH2	NPM1
Subsample 1	47.79	95.08	92.97	58.04	46.36
Subsample 2	51.72	91.23	91.67	59.62	47.45
Subsample 3	45.05	93.69	89.55	60.66	45.27
Subsample 4	42.11	89.92	92.31	53.85	41.77
Subsample 5	53.76	93.81	89.04	53.85	39.88
Subsample 6	50.00	90.35	93.18	46.67	42.86
Subsample 7	46.25	95.38	91.57	47.54	44.94
Subsample 8	61.84	89.23	95.89	51.06	45.51
Subsample 9	42.22	93.58	95.79	56.60	41.90
Subsample 10	47.78	93.86	92.00	57.69	41.13
Subsample 11	41.56	92.54	93.24	49.09	41.21
Subsample 12	43.68	91.82	91.18	56.25	42.86
Subsample 13	55.41	93.97	84.62	40.35	40.48
Subsample 14	48.86	91.04	89.53	63.16	37.50
Subsample 15	46.43	82.98	92.68	54.84	37.36
Mean	48.30	91.90	91.68	53.95	42.43
Std.	5.60	3.10	2.79	6.08	3.04

TABLE S2. Variation in VRF (%) for different subsamples of 10,000 concatenated reads for the Horizon myeloid reference (HD829).

Section S4: Analytical Validation Experiments with Synthetic DNA

Analytical validation experiments using synthetic DNA spike-in samples. Multi-gene OCEANS panels were calibrated by a spiking synthetic mutation-bearing DNA oligonucleotide (gBlock) into the human NA18562 gDNA. The 10% VAF positive control sample had its VAF confirmed by NGS, and was diluted with NA18562 gDNA to prepare positive control samples with VAF ranging between 1% and 0.05%. For 1% and 0.5% VAF samples, 50 ng input DNA was used for OCEANS panels, corresponding to 15,000 haploid copies. For 0.2%, 0.1%, and 0.05% VAF samples, 100 ng input DNA was used for OCEANS panels.

OCEANS reads were deconcatenated, aligned to human reference genome (GRCh38), and then both the Clair score and the Variant Read Fraction (VRF) were calculated for each mutation. A summary of the variant calls made by Clair for melanoma OCEANS panel's calibration runs is shown in Fig. S10. Chromosome positions covered by the OCEANS panels are shown in Table S3 to Table S6. Spike-in mutations tested and their computed median enrichment fold (EF) values for AML and melanoma panel are shown in Table S7 to Table S10.

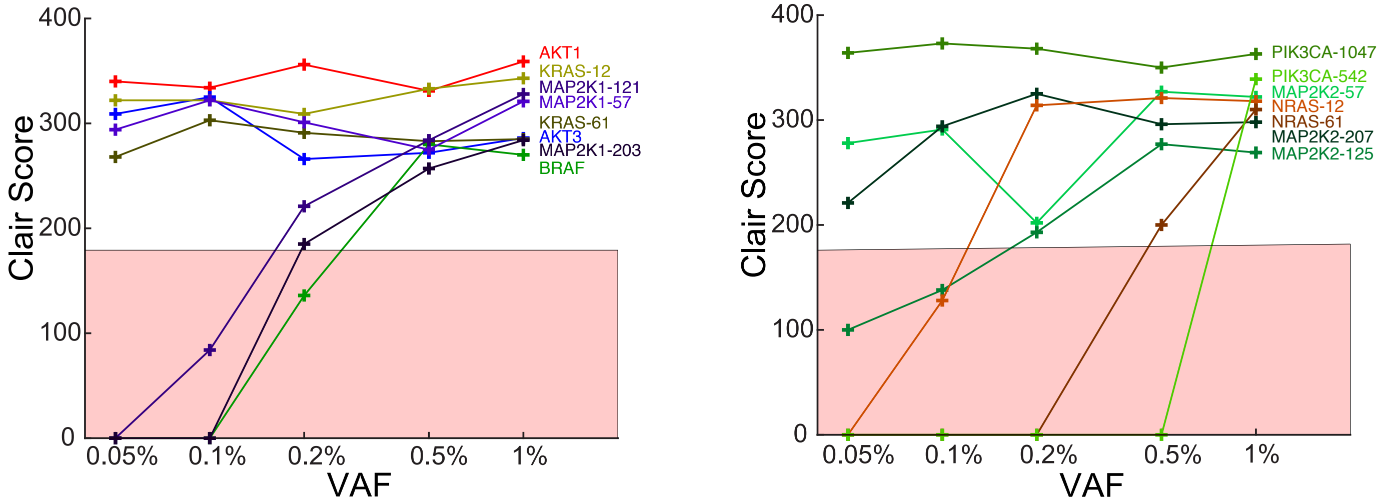

FIG. S10. Summary of Clair score for OCEANS melanoma panel. Mutations with Clair score > 180 are considered high confidence mutations.

Enrichment fold (EF) calculation. Enrichment fold was calculated as described previously in ref. [1]. Briefly, EF was calculated from the variant read fraction (VRF) observed from NS after OCEANS and the variant allele fraction (VAF) in the original sample based on the formula:

$$EF = \frac{(\frac{1-VAF}{VAF})}{(\frac{1-VRF}{VRF})}$$

For the AML and melanoma panels, EF was calculated for each plex by spiking synthetic DNA bearing a mutation into NA18562 gDNA shown in Fig. 4a and Fig. 4d. Median EF was calculated from EF values in the linear range from 0.05% to 1% VAF for each plex. Given a EF value for a particular mutation, the original VAF of the mutation in the sample can, in principle, be calculated from the VRF based on the formula $VAF = \frac{VRF}{(EF-1)(1-VRF)+1}$. However, due to the relatively high variation in observed VRF due to nanopore sequencing's intrinsic error rate, the effective dynamic range of quantitation is small, and VAF estimations are accurate only when the observed VRF is between 10% and 90%. For example, given an EF value of 1000, 99% VRF and 95% VRF correspond to VAFs of 10% and 2%. At 85% vs. 80% VRF, the VAFs correspond to 0.56% and 0.40%, which is a relatively smaller difference.

Mutation detection without SAL. AML OCEANS panel was run on 1% VAF synthetic DNA spike-in samples without SAL. Enrichment of variants were similar to OCEANS panel with SAL (Fig. S4-2). However, the throughput without SAL was around 6-fold lower. Therefore, BDA alone will be able to detect mutations as low as 1% VAF while SAL significantly improves throughput for mutation detection.

Gene	Enrichment Region (GRCh38)
FLT3	Chr13: 28,018,487-28,018,513
DNMT3A	Chr2: 25,234,363-25,234,383
IDH1	Chr2: 208,248,383-208,248,413
KIT	Chr4: 54,733,139-54,733,168
NPM1	Chr5: 171,410,531-171,410,557
IDH2	Chr15: 90,088,694-90,088,719
IDH2	Chr15: 90,088,599-90,088,623

TABLE S3. Chromosome positions enriched by the 7-plex OCEANS AML panel.

Gene	Enrichment Region (GRCh38)
MAP2K1	Chr15: 66,435,114-66,435,129
MAP2K1	Chr15: 66,436,814-66,436,830
MAP2K1	Chr15: 66,481,788-66,481,804
MAP2K2	Chr19: 4,117,540-4,117,558
MAP2K2	Chr19: 4,110,573-4,110,588
MAP2K2	Chr19: 4,101,089-4,101,106
AKT1	Chr14: 104,776,700-104,776,714
AKT3	Chr1: 243,695,699-243,695,726
NRAS	Chr1: 114,716,123-114,716,137
NRAS	Chr1: 114,713,894-114,713,912
KRAS	Chr12: 25,245,346-25,245,358
KRAS	Chr12: 25,227,328-25,227,346
PIK3CA	Chr3: 179,218,291-179,218,309
PIK3CA	Chr3: 179,234,293-179,234,307
BRAF	Chr7: 140,753,333-140,753,353

TABLE S4. Chromosome positions enriched by the 15-plex OCEANS melanoma panel.

Gene	Enrichment Region (GRCh38)
CTNNB1	Chr3: 41,224,607-41,224,626
CTNNB1	Chr3: 41,224,645-41,224,663
ARID1A	Chr1: 26,729,717-26,729,730
AXIN1	Chr16: 346,763-346,779
TERT	Chr5: 1,295,114-1,295,123
JAK1	Chr1: 64,845,513-64,845,528
PTEN	Chr10: 87,933,139-87,933,154
TP53	Chr17: 7,675,081-7,675,091
TP53	Chr17: 7,674,879-7,674,896
TP53	Chr17: 7,674,210-7,674,222
TP53	Chr17: 7,673,802-7,673,816

TABLE S5. Chromosome positions enriched by the 11-plex OCEANS HCC panel.

[1] Song, P., Chen, S. X., Yan, Y. H., Pinto, A., Cheng, L. Y., Dai, P., Patel, A. A., & Zhang, D. Y. Detecting and Quantitating Low Fraction DNA Variants with Low-Depth Sequencing. BioRxiv, 2020.04.26.061747 (2020).

Gene	Enrichment Region (GRCh38)
AKT1	Chr14: 104,780,200-104,780,218
ALK	Chr2: 29,222,334-29,222,352
ALK	Chr2: 29,220,830-29,220,847
ALK	Chr2: 29,213,992-29,214,009
ALK	Chr2: 29,209,816-29,209,832
BRAF	Chr7: 140,753,326-140,753,346
BRAF	Chr7: 140,781,595-140,781,614
DDR2	Chr1: 162,778,599-162,778,613
EGFR	Chr7: 55,174,001-55,174,015
EGFR	Chr7: 55,174,769-55,174,790
EGFR	Chr7: 55,181,309-55,181,322
EGFR	Chr7: 55,181,378-55,181,391
EGFR	Chr7: 55,191,817-55,191,831
KRAS	Chr12: 25,227,341-25,227,356
KRAS	Chr12: 25,245,340-25,245,352
KRAS	Chr12: 25,225,609-25,225,628
MAP2K1	Chr15: 66,435,106-66,435,124
MET	Chr7: 116,771,974-116,771,998
NRAS	Chr1: 114,716,111-114,716,127
NRAS	Chr1: 114,713,907-114,713,924
PIK3CA	Chr3: 179,218,294-179,218,311
PIK3CA	Chr3: 179,234,281-179,234,301
PTEN	Chr10: 87,957,911-87,957,922
ROS1	Chr6: 117,318,223-117,318,238
TP53	Chr17: 7,675,081-7,675,091
TP53	Chr17: 7,674,879-7,674,896
TP53	Chr17: 7,674,217-7,674,230
TP53	Chr17: 7,673,802-7,673,816

TABLE S6. Chromosome positions enriched by the 28-plex OCEANS NSCLC panel.

Gene	Mutation	Median EF
KIT	2446G>C	1420
FLT3	2504A>T	886
DNMT3A	2645G>A	32.4
IDH1	394C>T	1280
IDH2	418C>T	128
IDH2	515G>T	61.7
NPM1	863_864insTCTG	361

TABLE S7. Median Enrichment Fold (EF) values observed experimentally for the 7-plex AML OCEANS panel.

Gene	Mutation	Median EF
AKT1	235G>T	5650
AKT3	49G>A	478
BRAF	1799T>A	78.1
KRAS	34G>A	625
KRAS	180_181GA>TT	4220
MAP2K1	169A>G	534
MAP2K1	361A>T	221
MAP2K1	607G>A	202
MAP2K2	169T>G	811
MAP2K2	373A>T	192
MAP2K2	619G>A	2580
NRAS	34G>A	143
NRAS	181G>T	99.2
PIK3CA	1624G>A	447
PIK3CA	3139G>A	6570

TABLE S8. Median Enrichment Fold (EF) values observed experimentally for the 15-plex melanoma OCEANS panel.

Gene	Mutation	Median EF
AKT1	49G>A	95
ALK	3512T>A	168.6
ALK	3520T>G	40.7
ALK	3734T>G	63.5
ALK	3806G>C	176
BRAF	1799_1800delinsAT	296.2
BRAF	1406G>T	15.6
DDR2	2304T>A	105.4
EGFR	2156G>C	11.8
EGFR	2248G>C	21.2
EGFR	2303G>T	39.4
EGFR	2369C>T	154.3
EGFR	2582T>A	46.3
KRAS	183A>C	103.5
KRAS	35G>A	81.7
KRAS	436G>C	38.7
MAP2K1	167A>C	13.6
MET	3024_3028+7delAGAAGGTATATT	84.9
NRAS	34G>A	85.9
NRAS	181C>A	272.7
PIK3CA	1624G>A	14.8
PIK3CA	3140A>T	188
PTEN	697C>T	180.2
ROS1	5957C>T	178
TP53	524G>A	82.6
TP53	637C>T	17.3
TP53	733G>A	135.2
TP53	c.818G>A	587.2

TABLE S9. Median Enrichment Fold (EF) values observed experimentally for the 28-plex NSCLC OCEANS panel.

Gene	Mutation	Median EF
CTNNB1	98C>G	93.4
CTNNB1	133T>C	35
ARID1A	1210C>T	49.5
AXIN1	254G>A	770.9
TERT	promoter G>A	46.6
JAK1	2108G>T	421.8
PTEN	388C>A	855.7
TP53	524G>A	179.8
TP53	637C>T	409.2
TP53	747G>T	51.3
TP53	c.818G>A	693

TABLE S10. Median Enrichment Fold (EF) values observed experimentally for the 11-plex HCC OCEANS panel.

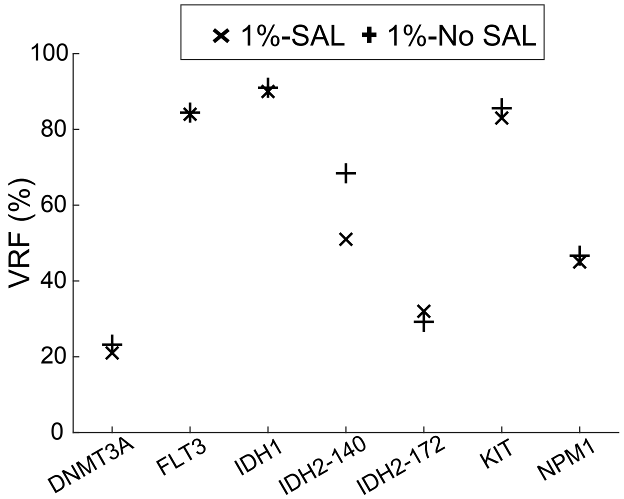

FIG. S11. AML OCEANS panel performance without SAL. Comparison of variant read frequencies (VRF) observed for 7-plex AML OCEANS panel with or without SAL. The input samples used here were internal reference samples constructed by spiking known quantities of synthetic DNA bearing a mutation of interest into NA18562 gDNA at 1% VAF. No significant differences were observed in VRF values without SAL. However, the throughput for the MinION run without SAL was only 106 Mb in one hour compared to 620 Mb in one hour with SAL.

Section S5. Mutations Detected in Melanoma Clinical Samples and NGS Comparison

Comparison of NS and Illumina NGS data on clinical samples. Fig. S12 shows comparison of OCEANS and Illumina NGS data separately for melanoma fresh frozen (FF) and FFPE clinical tissue samples. Mutations called by the OCEANS panel for melanoma, NSCLC and HCC clinical samples are listed in the excel file. Fig. S13 shows the location of TERT promoter mutation in a homopolymer region. FF and FFPE samples where insufficient amounts of DNA (< 5ng) were extracted were excluded from these lists, and OCEANS panels were not run on them. Table S11 shows clinical sample information for melanoma FF samples; FFPE sample clinical information were not available. Table S12 and Table S13 show clinical sample information for NSCLC and HCC FF and FFPE samples.

There are significantly higher number of mutations calls in FFPE samples compared to FF samples, such as the MAP2K1 c.371C>T mutation observed in 15 of the melanoma FFPE samples. The high concordance with digital PCR (Supplementary Section S6) generally suggests that these called variants are not due to systematic errors in the OCEANS method. A more likely explanation is that these mutations may have arisen from cytosine deamination DNA damage from FFPE treatment. This is supported by the fact that FFPE mutation calls had a significant over-representation of C>T and G>A mutations that derive from cytosine deamination.

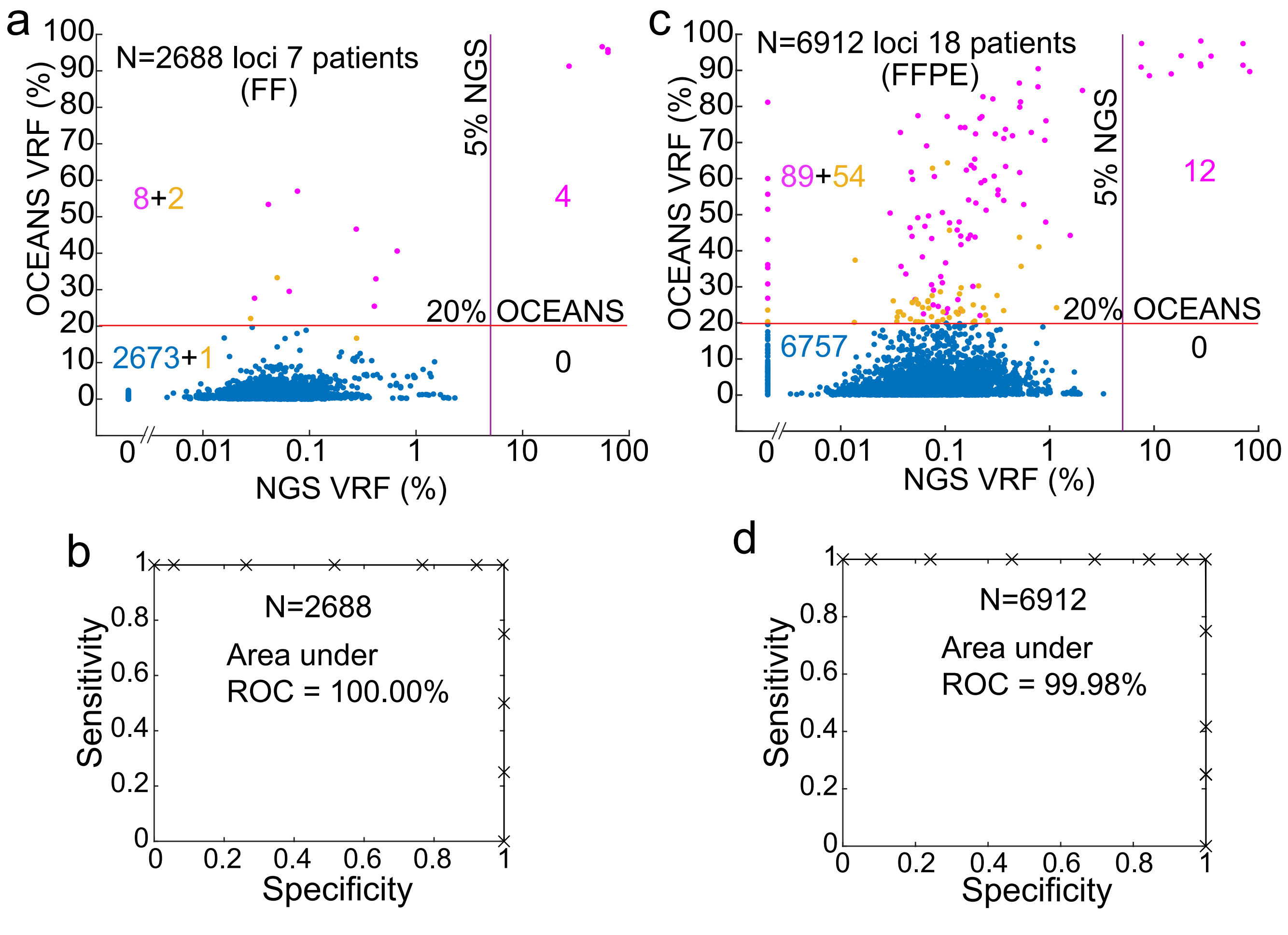

FIG. S12. Scatter plot comparison of NS and Illumina NGS data on melanoma clinical samples. **(a)** Comparison on fresh frozen clinical samples. %VRF for each nucleotide position in the enrichment region is plotted against the corresponding %VRF in NGS. Number of data points in each quadrant is indicated. 8 putative variants in the top-left quadrant had both VRF >20% and Clair score above 180 (purple dots). 3 putative variants had either VRF >20% or Clair score above 180 (yellow dots). **(b)** ROC curve for fresh frozen clinical samples. NGS inferred VAF > 5% were considered true positives. **(c)** Comparison on FFPE clinical samples. VRF for each nucleotide position in the enrichment region is plotted against the corresponding VRF in NGS. Number of data points in each quadrant is indicated. 89 putative variants in the top-left quadrant had both VRF >20% and Clair score above 180 (purple dots). 54 putative variants had either VRF >20% or Clair score above 180 (yellow dots). **(d)** ROC curve for FFPE clinical samples.

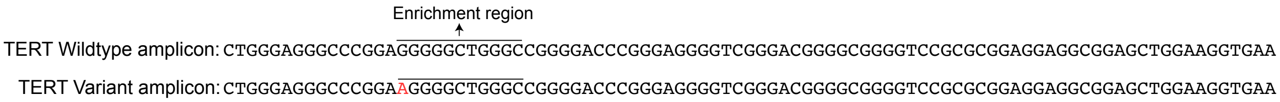

FIG. S13. TERT amplicon sequence in HCC OCEANS panel. G>A mutation is located within a homopolymer region in TERT promoter. Any variant in the underlined sequence is enriched by BDA.

Sample	Stage	Age	Gender	Pathology
FF172	IV	66	Female	Malignant melanoma
FF3	IV	42	Female	Malignant melanoma
FF52	IV	66	Male	Malignant melanoma
FF26	IV	50	Male	Malignant melanoma
FF61	IV	42	Female	Malignant melanoma
FF2	IV	46	Female	Malignant melanoma
FF20	II	57	Male	Malignant melanoma

TABLE S11. Melanoma fresh/frozen clinical sample information.

Sample	Stage	Age	Gender	Pathology
FFPE1	IV	70	Male	Adenocarcinoma of lung
FFPE2	IB	47	Female	Adenocarcinoma of lung
FFPE3	IIB	66	Female	Adenocarcinoma of lung
FFPE4	IB	73	Female	Carcinoma of lung, non-small cell
FFPE5	IB	78	Male	Carcinoma of lung, squamous cell
FFPE6	IIB	77	Male	Carcinoma of lung, squamous cell
FFPE7	IB	64	Female	Adenocarcinoma of lung
FFPE8	IIIA	78	Male	Carcinoma of lung, squamous cell
FFPE9	IB	87	Male	Adenocarcinoma of lung, acinar, papillary
FFPE10	IB	76	Male	Carcinoma of lung, squamous cell
FFPE11	IB	68	Male	Carcinoma of lung, squamous cell
FFPE12	IA	62	Female	Carcinoma of lung, squamous cell
FFPE13	IIB	66	Female	Adenocarcinoma of lung
FFPE14	IIIA	62	Male	Carcinoma of lung, squamous cell
FFPE15	IB	75	Female	Carcinoma of lung, squamous cell
FFPE16	IIB	79	Female	Adenocarcinoma of lung
FFPE17	IB	64	Male	Adenocarcinoma of lung
FFPE18	IIIA	66	Male	Adenocarcinoma of lung, papillary
FF19	IA	74	Male	Adenocarcinoma of lung
FF20	IA	59	Female	Adenocarcinoma of lung
FF21	IA	62	Female	Carcinoma of lung, squamous cell
FF22	IV	52	Male	Adenocarcinoma of lung
FF23	IIIA	78	Male	Carcinoma of lung, squamous cell

TABLE S12. NSCLC clinical sample information.

Sample	Stage	Age	Gender	Pathology
FFPE1	IIIB	65	Male	Hepatocellular carcinoma
FFPE2	IB	47	Male	Hepatocellular carcinoma
FFPE3	IB	59	Female	Hepatocellular carcinoma
FFPE4	II	70	Male	Hepatocellular carcinoma
FFPE5	IIIA	49	Male	Hepatocellular carcinoma
FFPE6	IB	60	Male	Hepatocellular carcinoma
FFPE7	IIIB	41	Female	Hepatocellular carcinoma
FFPE8	IVA	51	Female	Hepatocellular carcinoma
FFPE9	IB	50	Male	Hepatocellular carcinoma
FFPE10	NA	40	Male	Hepatocellular carcinoma
FFPE11	I	51	Male	Hepatocellular carcinoma
FFPE12	NA	44	Male	Hepatocellular carcinoma
FFPE13	NA	62	Male	Hepatocellular carcinoma
FFPE14	NA	67	Male	Hepatocellular carcinoma
FFPE15	IIIA	68	Male	Hepatocellular carcinoma
FFPE16	I	73	Male	Hepatocellular carcinoma
FF17	II	68	Male	Hepatocellular carcinoma
FF18	IIIA	86	Male	Hepatocellular carcinoma
FF19	IIIA	81	Male	Hepatocellular carcinoma
FF20	IIIA	71	Male	Hepatocellular carcinoma
FF21	II	63	Male	Hepatocellular carcinoma

TABLE S13. HCC clinical sample information.

Section S6: ddPCR Comparison Experiments

We performed a total of 24 ddPCR comparison experiments on 6 FFPE DNA samples, testing 4 mutations each (BRAF p.V600E, KRAS p.G13D, KRAS p.E62K, and MAP2K1 p.P124L). We observe that 21 of the 24 matched experiments gave concordant results, with 10 of these concordant results being concordant positives. 2 of the 11 concordant negative values were deemed OCEANS negative because they only satisfied one of the two criteria (based on VRF and Clair), suggesting that the orthogonal bioinformatic validation improved variant call accuracy.

Individual ddPCR results and comparison to OCEANS % VRF are shown in figures S14, S15, S16 and S17. Green dots are HEX (WT) positive droplets, blue dots are FAM (Variant) positive droplets, red dots are double positive, and black dots are negative droplets. VAF was calculated as VAF = ((Variant positive+Double positive)/(WT positive + Variant positive+Double positive)).

Sample	BRAF p. V600		KRAS p. G13D		KRAS p. E62K		MAP2K1 p. P124L	
	OCEANS	ddPCR VAF	OCEANS	ddPCR VAF	OCEANS	ddPCR VAF	OCEANS	ddPCR VAF
FFPE3	Yes	31.13%	Yes	0.03%	No	0%	Yes	0.28%
FFPE13	Yes	0.66%	No*	0%	No	0%	Yes	0.15%
FFPE17	No	0%	Yes	0%	No	0.43%	No	0%
FFPE18	No	0%	Yes	0.04%	No	0%	No	0.50%
FFPE19	No	0%	No*	0%	Yes	0.05%	Yes	0.64%
FFPE25	No	0%	No	0%	Yes	0.02%	Yes	0.14%
FF3	Yes	0.33%	NA	NA	NA	NA	NA	NA

TABLE S14. Summary of ddPCR results for BRAF p.V600E, BRAF p.V600K, KRAS p.G13D, KRAS p.E62K, MAP2K1 p.P124L mutations in melanoma clinical samples. Concordant positive results are displayed in green, concordant negative results in blue, and discordant results in red. *OCEANS variant call satisfied one condition for a variant call, either VRF > 20% or Clair score >180.

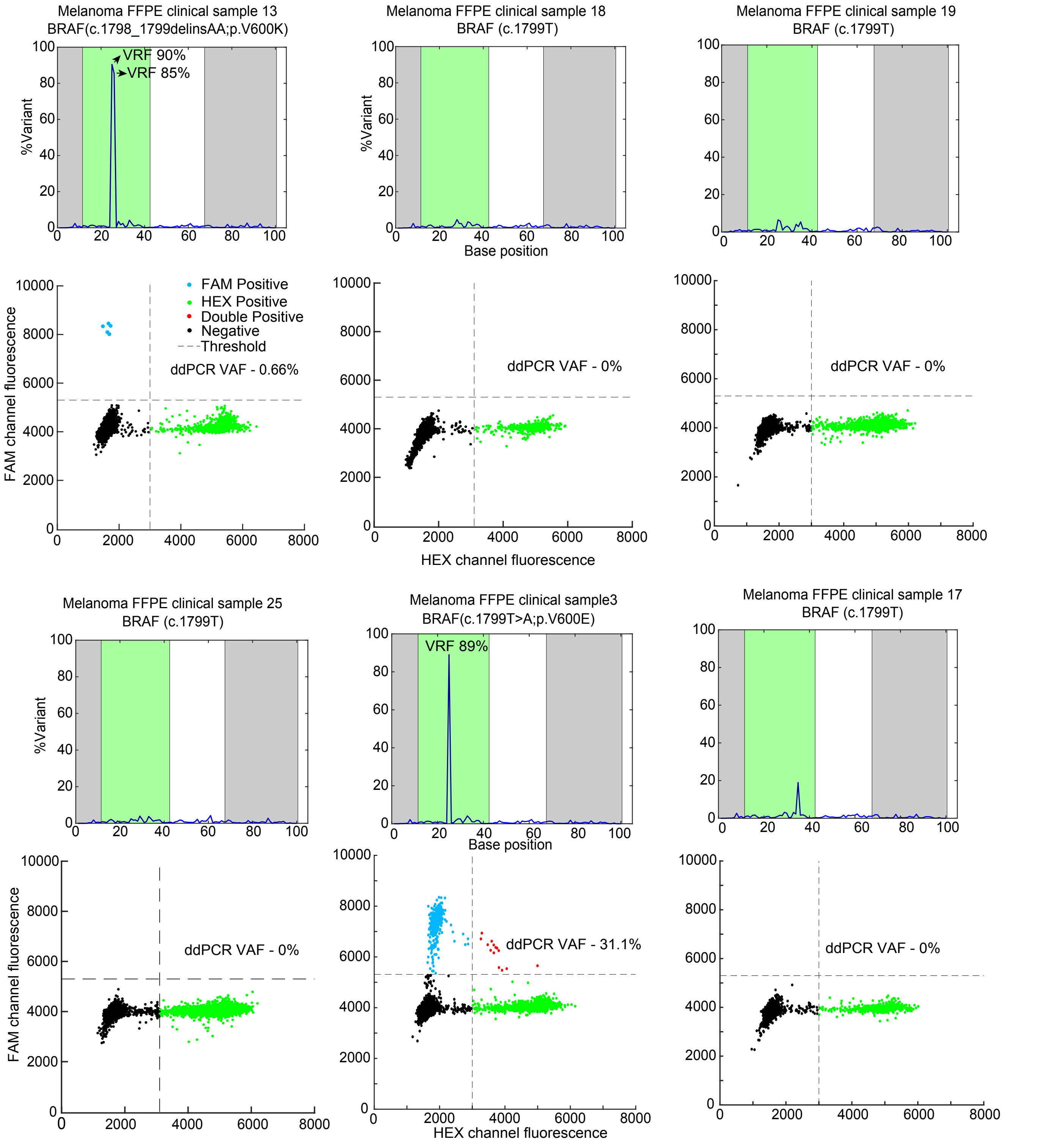

FIG. S14. NS and ddPCR comparison for mutation at BRAF (c.1799T) position in melanoma clinical samples. Top panel shows OCEANS results. Forward and reverse primer regions are shaded in gray, and the BDA enrichment region is shaded in green. Bottom panel shows ddPCR results for the same sample.

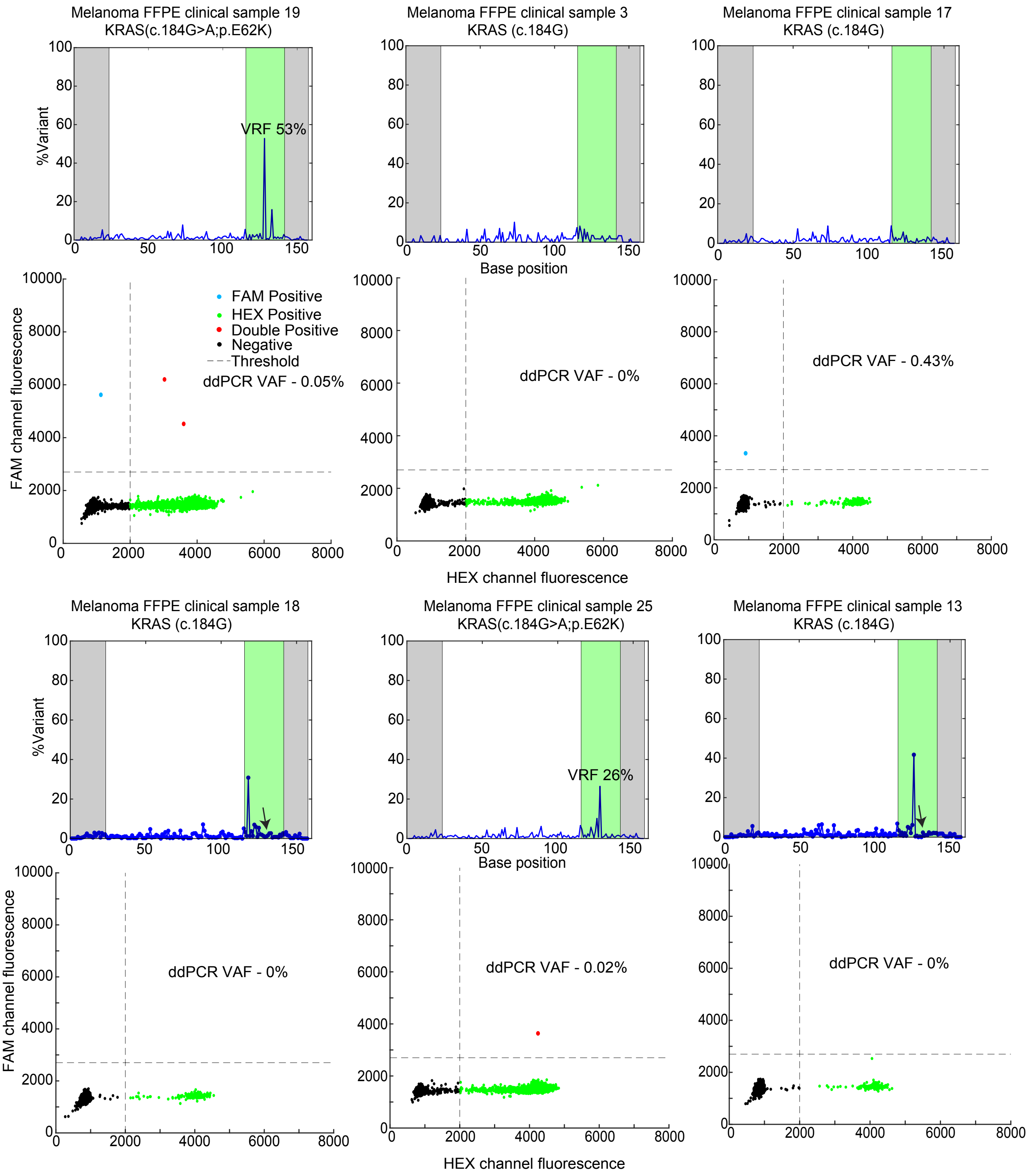

FIG. S15. NS and ddPCR comparison for mutation at KRAS (c.184G) position in melanoma clinical samples. Top panel shows OCEANS results. Forward and reverse primer regions are shaded in gray, and the BDA enrichment region is shaded in green. Bottom panel shows ddPCR results for the same sample. For FFPE sample 18 and 13, the c.184 position is indicated by arrows.

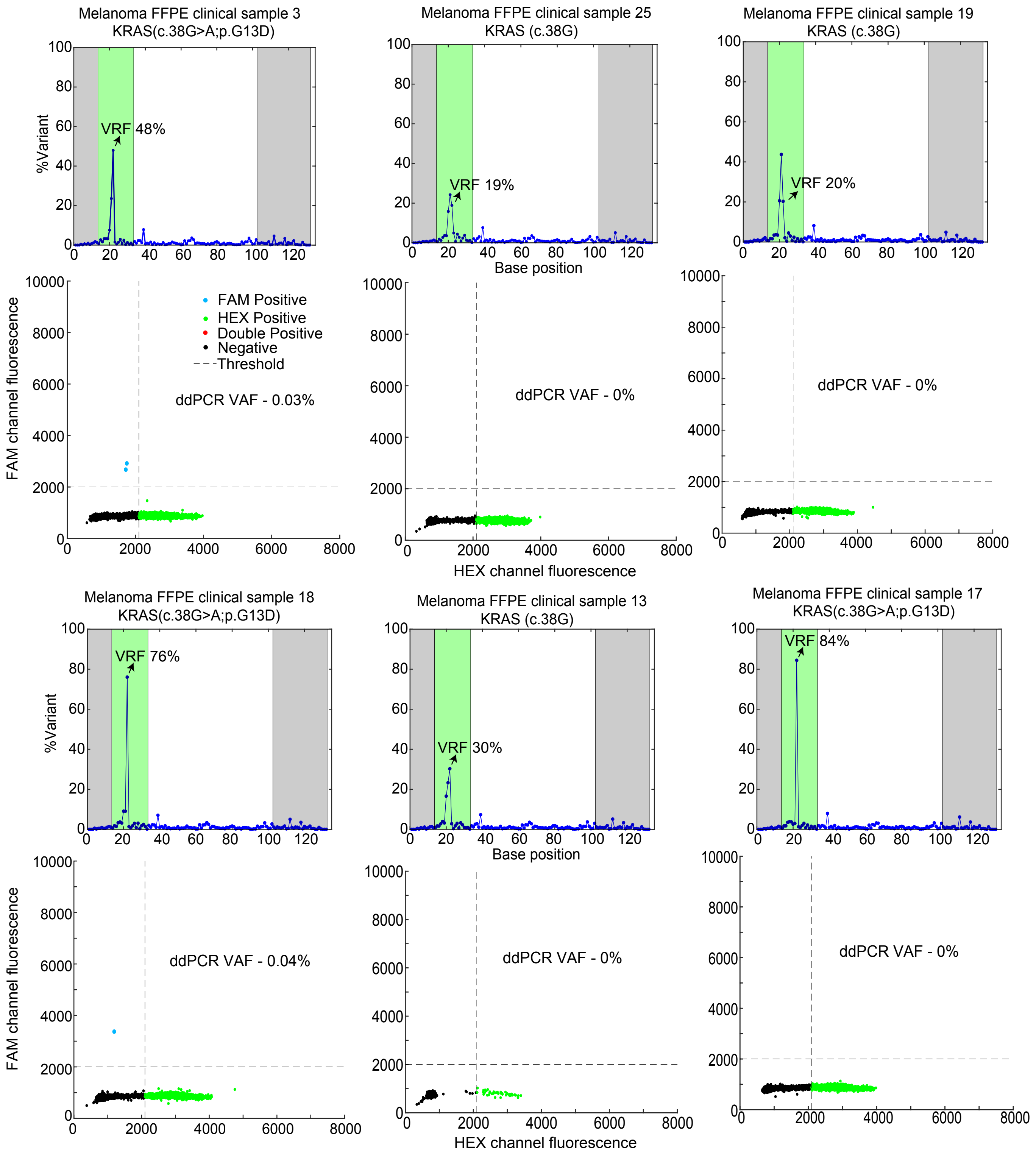

FIG. S16. NS and ddPCR comparison for mutation at KRAS (c.38G) position in melanoma clinical samples. Top panel shows OCEANS results. Forward and reverse primer regions are shaded in gray, and the BDA enrichment region is shaded in green. Bottom panel shows ddPCR results for the same sample. The c.38 position is indicated by arrows. The % VRF at this loci was high for all samples tested, but Clair scores were ¿180 only for FFPE samples 3, 18 and 17.

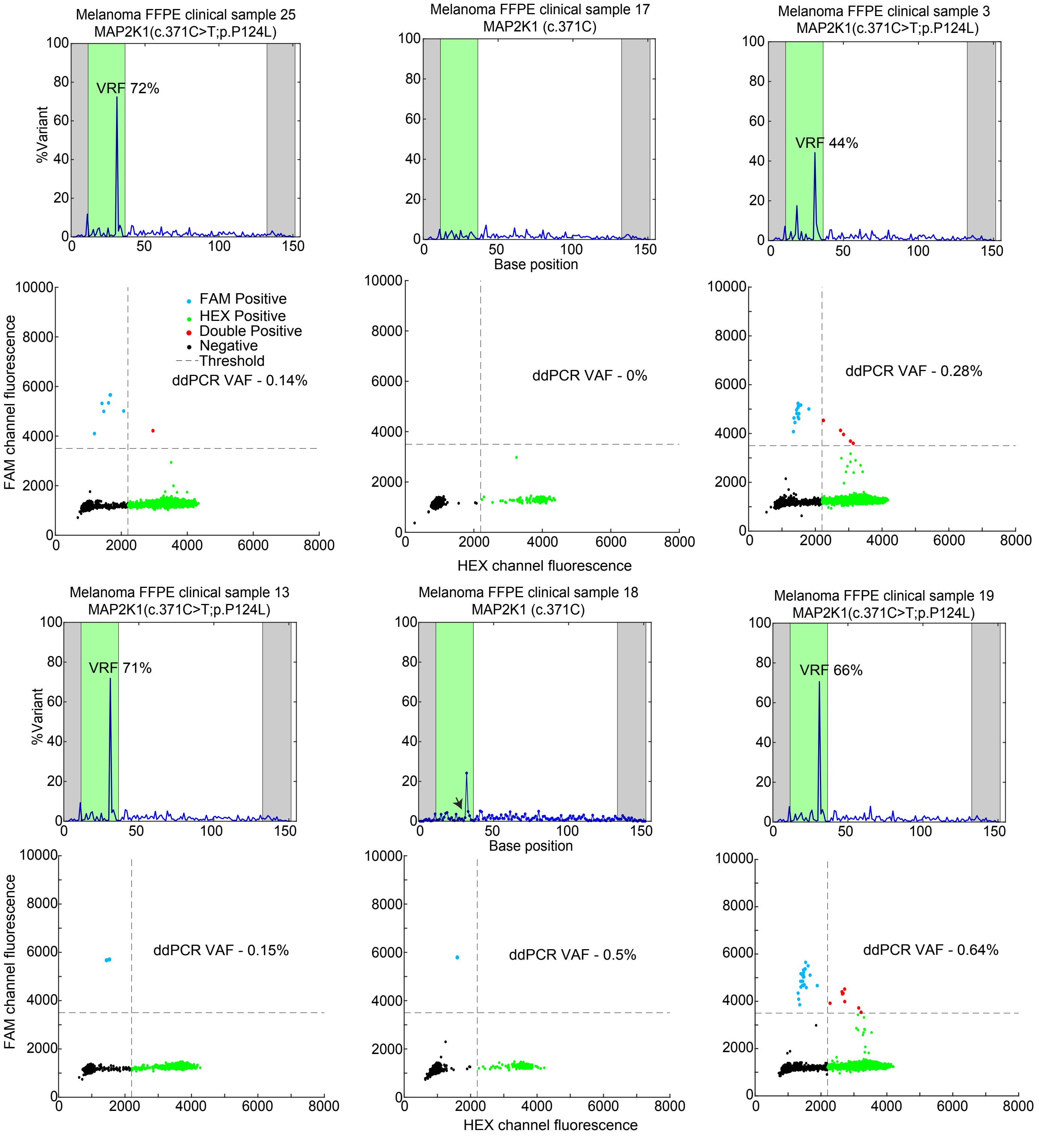

FIG. S17. NS and ddPCR comparison for mutation at MAP2K1 (c.371C) position in melanoma clinical samples. Top panel shows OCEANS results. Forward and reverse primer regions are shaded in gray, and the BDA enrichment region is shaded in green. Bottom panel shows ddPCR results for the same sample. The c.371 position is indicated by arrow for FFPE sample 18.
