# Supplementary material for: Oncogene Concatenated Enriched Amplicon Nanopore Sequencing for rapid, accurate, and affordable somatic mutation detection
Source: Genome Biol. 2021 Sep 6;22:227. doi: 10.1186/s13059-021-02449-1 (PMC8419911; doi:10.1186/s13059-021-02449-1)
Supplement: Supplementary file 6 — Additional file 6 Review history [file 13059_2021_2449_MOESM6_ESM.docx]

Review History

**First round of review**

**Reviewer 1**

**Were you able to assess all statistics in the manuscript, including the appropriateness of statistical tests used?** Yes: No additional review is required on the statistics.

**Were you able to directly test the methods?** No

**Comments to author:**

Thirunavukarasu et al. present "Oncogene Concatenated Enriched Amplicon Nanopore Sequencing for Rapid, Accurate, and Affordable Somatic Mutation Detection" in which a new technique "OCEANS" is described, which combines BDA and an adapted Golden gate assembly method (called SAL) to measure cosmic mutations using nanopore sequencing. The authors first nicely show that SAL is required for a higher throughput that would be sufficient for applications in the clinic. Then, BDA is leveraged to increase the sequencing quality that is inherently lower in nanopore sequencing. OCEANS allows the detection of mutations with low VAF in several tumor types, due to a massive enrichment of the mutated allele specifically. However, in spite of the efforts of the authors to create a technique that is suitable for the clinic, a major limitation of the approach, which arguably would preclude clinical utility, is that only an present/absent call is obtained rather than an estimate of the VAF. Furthermore, I have concerns about the high number of observed mutations in OCEANS that are missed in NGS, and are assumed to be true positives based on a very low number of ddPCR experiments. The paper is well written, certainly has merit, but, in my opinion, requires revision on the following points:

Major comments

1 - A major drawback of the block displacement amplification (BDA) is that it is difficult to infer the initial VAF of the mutated allele. On top of the BDA, the authors also perform a size selection after SAL, making the VAFs even less reliable. Consequently, OCEANS can only detect whether a variant is present/absent in a sample. Unfortunately, in spite of the efforts of the authors to enable clinical application, this renders the technique largely unsuitable for the clinic as prognostic use or use for treatment selection will require information on the VAF to prevent making decision based on a mutation that could only be present in <1% of the tumor cells. For early detection in a liquid biopsy setting, it is well-known that hematopoietic cells harbour mutations that may be confused to be tumor mutations and this problem may be amplified using OCEANS. For minimal residual disease (MRD) detection, the technique has value, but it will still be important to show the VAF of a mutation instead of a simple yes/no verdict.

Looking at the mutations that are missed by NGS and checked in ddPCR, I notice that only one of these mutations has a VAF > 0.7%, suggesting that these mistakes may be more often than desired and may confuse final diagnosis. The authors should test experimentally whether the solution that they provide in the discussion (usage of UMIs) will indeed resolve this problem.

2 - The ROC curves and sensitivity/specificity calculations in this manuscript are misleading. The ROC curves contain results of all cosmic positions in the reads of all patients, which are mostly true negatives. Information of false negative and false positive are diluted by the true negative positions that are less of interest. The authors should focus or at least include precision / recall.

3 - I have concerns about the false positive rate. On page 7, the authors mention that they "believe that many of the 97 discordant called variants based on a 20% VRF threshold and a Clair score ≥180 could be real mutations missed by NGS, based on our calibration experiments". They also confirm this for 10/11 mutations at 4 mutation loci using ddPCR. From these results they conclude that "standard NGS has a somatic mutation sensitivity of 14.2%". However, the ddPCR locations should be chosen randomly if the authors want to make claims about the false negative rate of NGS (and the true positive rate of OCEANS). Given the fact that 11 (of 97) mutations were selected in only 4 of the 384 loci, this does not seem to be the case and, therefore, the other 85 mutations could still largely be false positives of OCEANS. Furthermore, 11 mutations is a very low number to determine the false positive rate. The authors should at least check all 97 loci in ddPCR. If the false positive rate is much lower when all of these regions are taken into account, the authors should also check random positions for the other tumor types to get a good estimate of the false positive rate.

4 - On page 11 it is mentioned that "Custom bioinformatics software were written in python for NS reads process and are available upon request. Nanopore raw FAST5 and FASTQ data are available upon request." The authors should make all code and data available according to the Genome Biology policy: https://genomebiology.biomedcentral.com/submission-guidelines/preparing-your-manuscript/research. As the authors extensively build upon publicly available bioinformatics software (e.g. Clair, minimap2, IGV, etc) I think it is only fair to share the code and data used in this work so that others can readily reuse and extend this work.

5 - The authors nicely show that SAL alone cannot be used for mutation detection at 5% VAF and that a combination of BDA and SAL can be used to detect very low VAFs. However, it is unclear whether BDA alone would be sufficient to detect mutations at e.g. 5%VAF.

Minor comments

1 - NS is not a common abbreviation for nanopore sequencing, and for readability it would be better to choose an abbreviation that is more distinct from NGS.

2 - Several more references to literature could be made. To give just one example: "with typical commercial NGS kits and services claiming LoDs of between 0.1% and 0.5% VAF." should include a reference.

3 - Some abbreviations are introduced twice (e.g. NS) while others are not introduced (e.g. SPRI). Please check this throughout the manuscript.

4 - Page 3: "In addition to improving the throughput of NS, we also found that the SAL reduced the quality/error rate of NS (Fig. 2d). The NS results of a 340 nt amplicon had a mean phred quality score of 9.87, corresponding to an error rate of 10.3%. The concatemer, in contrast, had a mean phred score of 11.55, corresponding to an error rate of 7.0%." This is most likely due to the fact that the ends contain more sequencing errors in nanopore reads, I presume. And you obviously have less ends with concatenated reads. Clipping of the ends of the reads may improve the sequencing quality even further. Have the authors tried this? Furthermore, if you clip off the ends of the short reads, is there still a difference between short and SAL fragments?

5 - p3,r36: "Simulations and prior literature [19] suggest" seems to suggest these simulations are carried out by the authors. If so, they should be included, otherwise this phrasing is misleading.

6 - "Variant calls were made using two different approaches: (1) based on the variant read frequency exceeding a threshold of 20%, and (2) based on a Clair [21] score of above 180." Why were these cutoffs chosen? If they are chosen based on the calibration experiments mentioned in the caption of Fig 4 and in Supplementary Section S4, I suggest moving the caption to the main text, and including the calibration experiments in the method section.

7 - The x labels and the y axis of Figure 4b should be aligned to Figure 4a.

8 - In the caption of Figure 5b the authors stated that 5 mutants in the panels all have supporting reads from NGS. However, we only see 4 arrows pointing towards mutations in fig 5b.

9 - The comparison in table 2 needs to be adjusted. Costs of the sample preparations (and multiplexing of samples) seem to be missing. The authors say you can reuse a flow cell 10 times, while ONT themselves say that you can only do this up to 5 times. Furthermore, the costs for flushing the flow cells are also missing from the calculations. In addition, the authors should add a row stating whether you measure the VAF of a mutation, or presence/absence of a mutation.

10 - In general I find the comparison between NGS and 'NS' in the introduction and in table 2 too opportunistic. The worst statistics are shown for NGS (e.g. not all machines take several days for sequencing), while for NS they mention uncommonly short sequencing times of fifteen minutes. Sure the main points are mostly correct, but it can be described a bit more realistic.

**Reviewer 2**

**Were you able to assess all statistics in the manuscript, including the appropriateness of statistical tests used?** Yes: No further review required.

**Were you able to directly test the methods?** No

**Comments to author:**

In the presented manuscript a new protocol for accurate Nanopore Sequencing (NS) and subsequent detection of somatic mutations with VAS limits of detection (LoD) between 0.05% and 1%.

The paper is very well written and easy to follow.

The challenges for somatic SNP detection in low VAF NS data are clearly outlined, and a very detailed and comprehensive approach is taken to explain the novel protocol OCEANS, main features of which combine:

* Stochastic Amplicon Ligation (SAL) which allows for creation of artificial longer fragments from shorter underlying DNA fragments, as to take advantage of the fact that NS platform throughput in the first hour of sequencing is mostly driven by the number of sequenced molecules, rather than their length

* Blocker Displacement Amplification (BDA) which allow for enrichment of the mutation-burdened clone/allele in the analyzed tumor samples, which can be heterogeneous in nature, thus limiting the VAF for the mutation(s) of interest.

Comprehensive proof-of-concept testing of the proposed OCEANS protocol was carried, and the results are presented side-by-side the gold-standard NGS sequencing output. Proposed methodology shows comparative performance to the NGS results, while providing a sizable reduction in cost and turnover time, which are of great importance in clinical application settings.

While the paper, as I've mentioned before, is well structured and very nicely written, a brief outline of the paper structure at the end of the introduction section can benefit the reader as to provide guidance that, first, the SAL component of the protocol if described with the T+N data analyzed, and then the BDA part follows. It would also improve clarity if the explicit statement was made for the described SAL approach w.r.t. NS, as to whether this is suitable for any short-fragment library (e.g., FFPE data genome-wide, cfDNA, etc) or if some targeting is needed/recommended.

All illustration in the paper are of high quality and clearly demonstrate all the described concepts and/or results, providing a reader with a comprehensive visual aid.

Page 2: Some clarification on why the SAL-prepared longer fragments/reads resulted in better quality/error rate of NS will benefit the reader. Is this intrinsic to the sequencing process, or basecalling, or something else?

Page 4: through the course of many PCR cycles --> some quantification of "many" is going to beneficial to the reader.

end of page 7, beginning of page 8: check VAR vs VRF correctness.

Page 10: the mentioned limitation/non-suitability of cfDNA and FFPE samples for NS processing and subsequent detection of "large-scale" alterations seems, from the outside, overoptimistic. Is there any reason why the proposed direct or SAL-based processing of FFPE and/or cfDNA can not be used for counting statistics detection, based on coverage profiles, of large-fragment, or even whole-chromosome CNV events? If not -- the reader will benefit from learning why. If possible, even in theory, this can be of tremendous interest to the community, both academic and clinical, as the turnaround time with NS is much better than with NGS.

Page 10: there may be a benefit of adding to the discussion mentioned about most recent progress of ONT small variant calling methodologies PEPPER/DeepVariant, and how they may improve th specificity/sensitivity of the proposed protocol, or if they are not really suitable for this application.

Overall, this is a very nicely written paper with a great science behind it. I strongly recommend this paper for publication after a minor revision, which would be better referred to as "polishing".

Minor comments:

* page 1, introduction: (VAF) remaining challenging --> (VAF) remains

* page 6, Fig 4, panels c and e: either add (5) to Y axis label, or to Y axis values.

**Authors Response**

**Point-by-point responses to the reviewers’ comments:**

Reviewer 1:

1 - A major drawback of the block displacement amplification (BDA) is that it is difficult to infer the initial VAF of the mutated allele. On top of the BDA, the authors also perform a size selection after SAL, making the VAFs even less reliable. Consequently, OCEANS can only detect whether a variant is present/absent in a sample. Unfortunately, in spite of the efforts of the authors to enable clinical application, this renders the technique largely unsuitable for the clinic as prognostic use or use for treatment selection will require information on the VAF to prevent making decision based on a mutation that could only be present in <1% of the tumor cells. For early detection in a liquid biopsy setting, it is well-known that hematopoietic cells harbour mutations that may be confused to be tumor mutations and this problem may be amplified using OCEANS. For minimal residual disease (MRD) detection, the technique has value, but it will still be important to show the VAF of a mutation instead of a simple yes/no verdict.

Thanks for pointing out this shortcoming. As we described in supplementary section S4, the VAF of the mutation can be calculated by OCEANS method, but with limited dynamic range. However, we agree with the reviewer, showing the VAF will be important for clinical utility. Therefore, we calculated the original sample VAFs from OCEANS VRF for all 3 of our panels and compared them to NGS VAFs. These were included as additional Figures 5d, 6c and 6d. Even though the OCEANS VRF saturates at high sample VAF, we can identify the high and low VAF mutations, which will aid in treatment selection.

SAL randomly concatenates amplicons into concatamers. Therefore, performing size selection should not significantly change the ratio of wild type to variant sequences in the library.

Looking at the mutations that are missed by NGS and checked in ddPCR, I notice that only one of these mutations has a VAF > 0.7%, suggesting that these mistakes may be more often than desired and may confuse final diagnosis. The authors should test experimentally whether the solution that they provide in the discussion (usage of UMIs) will indeed resolve this problem.

We chose mutations with NGS VAF around 1% to be confirmed by the more sensitive ddPCR method. We included the OCEANS calculated VAFs, NGS VAF and ddPCR VAFs for these mutations in supplementary excel table. There is generally good agreement in terms of the VAF values between the three methods except for one del-ins mutation. This can be explained by the difference in enrichment fold between the observed mutation and that used in calibration experiments. The calculated OCEANS VAFs will prevent confusion in the final diagnosis for such low VAF mutations. As the reviewer suggested, the use of UMIs will help to resolve the quantitation problem with the OCEANS method by expanding the dynamic range of quantitation. But low VAF <1% is within the dynamic range of quantitation by the OCEANS method. Higher VAFs are more apparent by high OCEANS VRF. Therefore, our method will

provide useful information for clinical diagnosis. Moreover, due to the higher error rate of Nanopore Sequencing, the integration of UMIs in OCEANS will require extensive experimental validation and bioinformatic work. Therefore, we will incorporate them in a future work.

2 - The ROC curves and sensitivity/specificity calculations in this manuscript are misleading. The ROC curves contain results of all cosmic positions in the reads of all patients, which are mostly true negatives. Information of false negative and false positive are diluted by the true negative positions that are less of interest. The authors should focus or at least include precision / recall.

Thank you for pointing out the missing data. We have included the precision/recall plots based on the calculated VAFs for all 3 panels in Figure 5f, 6g and 6h.

3 - I have concerns about the false positive rate. On page 7, the authors mention that they ”believe that many of the 97 discordant called variants based on a 20% VRF threshold and a Clair score >180 could be real mutations missed by NGS, based on our calibration experiments”. They also confirm this for 10/11 mutations at 4 mutation loci using ddPCR. From these results they conclude that ”standard NGS has a somatic mutation sensitivity of 14.2%”. However, the ddPCR locations should be chosen randomly if the authors want to make claims about the false negative rate of NGS (and the true positive rate of OCEANS). Given the fact that 11 (of 97) mutations were selected in only 4 of the 384 loci, this does not seem to be the case and, therefore, the other 85 mutations could still largely be false positives of OCEANS. Furthermore, 11 mutations is a very low number to determine the false positive rate. The authors should at least check all 97 loci in ddPCR. If the false positive rate is much lower when all of these regions are taken into account, the authors should also check random positions for the other tumor types to get a good estimate of the false positive rate.

The limit of detection of standard NGS is around 2% to 5% VAF. As our calibration experiments and subsequent ddPCR experiments show, OCEANS can detect mutations as low as 0.1% VAF. So, Figure 5c is not a fair comparison to infer false positive rate for OCEANS. We estimated the original sample VAF from OCEANS VRF and compared it to NGS VRF in a new Figure 5d. In this new figure, it can be seen that the all but one of the 97 discordant variants have an estimated VAF <5%. In Figure 5f, the AUC for precision-recall is 97.54%, which shows a low false positive rate for OCEANS compared to NGS. The purpose of the ddPCR experiments was not to determine the false negativity rate of NGS but to support the detection of low VAF mutations by OCEANS. Considering the limited availability of clinical samples, the cost and time involved in designing and performing ddPCR for all 97 loci, we have removed the statement “standard NGS has a somatic mutation sensitivity of 14.2%” in the revised manuscript.

4 - On page 11 it is mentioned that ”Custom bioinformatics software were written in python for NS reads process and are available upon request. Nanopore raw FAST5 and FASTQ data are available upon request.” The authors should make all code and data available according to the Genome Biology policy: <https://genomebiology.biomedcentral.com/submission-guidelines/preparing-your-manuscript/research>. As the authors extensively build upon publicly available bioinformatics software (e.g. Clair, minimap2, IGV, etc) I think it is only fair to share the code and data used in this work so that others can readily reuse and extend this work.

Thank you for pointing out the data availability requirements. The bioinformatics software for Nanopore reads processing was made available through Github. Nanopore and NGS sequencing data was deposited in NCBI Sequence Read Archive. Detailed SRA accessions were included in the supplementary material.

5 - The authors nicely show that SAL alone cannot be used for mutation detection at 5% VAF and that a combination of BDA and SAL can be used to detect very low VAFs. However, it is unclear whether BDA alone would be sufficient to detect mutations at e.g. 5%VAF.

Thanks for pointing out the missing data. BDA alone can detect 1% VAF. But without SAL, the throughput of shorter reads is lower. We included this data in supplementary section S4.

Minor comments 1 - NS is not a common abbreviation for nanopore sequencing, and for readability it would be better to choose an abbreviation that is more distinct from NGS.

We have replaced NS with Nanopore Sequencing, since we could not find a commonly used abbreviation for Nanopore Sequencing.

2 - Several more references to literature could be made. To give just one example: ”with typical commercial NGS kits and services claiming LoDs of between 0.1% and 0.5% VAF.” should include a reference.

We have included the references.

3 - Some abbreviations are introduced twice (e.g. NS) while others are not introduced (e.g. SPRI). Please check this throughout the manuscript.

Thanks for pointing out the error. We have corrected the error.

4 - Page 3: ”In addition to improving the throughput of NS, we also found that the SAL reduced the quality/error rate of NS (Fig. 2d). The NS results of a 340 nt amplicon had a mean phred quality score of 9.87, corresponding to an error rate of 10.3%. The concatemer, in contrast, had a mean phred score of 11.55, corresponding to an error rate of 7.0%.” This is most likely due to the fact that the ends contain more sequencing errors in nanopore reads, I presume. And you obviously have less ends with concatenated reads. Clipping of the ends of the reads may improve the sequencing quality even further. Have the authors tried this? Furthermore, if you clip off the ends of the short reads, is there still a difference between short and SAL fragments?

According to ONT technical support, the lower quality score of shorter reads is due to lack of sufficient current signal information for proper normalization prior to basecalling by MinKNOW. We used barcoded reads for both SAL and short fragments, which have 34 nucleotides as barcode on both ends. The barcodes were clipped before analysis, so clipping off the ends of short reads doesn’t account for the difference in quality. We have included this explanation in the manuscript.

5 - p3,r36: ”Simulations and prior literature [19] suggest” seems to suggest these simulations are carried out by the authors. If so, they should be included, otherwise this phrasing is misleading.

Thanks for pointing out the error. We have removed the word “simulations” in the revised version.

6 - ”Variant calls were made using two different approaches: (1) based on the variant read frequency exceeding a threshold of 20%, and (2) based on a Clair [21] score of above 180.” Why were these cutoffs chosen? If they are chosen based on the calibration experiments mentioned in the caption of Fig 4 and in Supplementary Section S4, I suggest moving the caption to the main text, and including the calibration experiments in the method section.

The thresholds were chosen based on internal work done at Oxford Nanopore Technologies and verified in our calibration experiments included in Figure 4 and Supplementary Figure S4-1.

7 - The x labels and the y axis of Figure 4b should be aligned to Figure 4a.

Thank you for the suggestion. However, the x labels of Figure 4b is not aligned to Figure 4A for clarity in labeling the individual traces in Figure 4b. Since, two of the traces start from 0 in Figure 4b, they cannot be labeled the same way as Figure 4a.

8 - In the caption of Figure 5b the authors stated that 5 mutants in the panels all have supporting reads from NGS. However, we only see 4 arrows pointing towards mutations in fig 5b.

Thanks for pointing out the error. We have corrected the error.

9 - The comparison in table 2 needs to be adjusted. Costs of the sample preparations (and multiplexing of samples) seem to be missing. The authors say you can reuse a flow cell 10 times, while ONT themselves say that you can only do this up to 5 times. Furthermore, the costs for flushing the flow cells are also missing from the calculations. In addition, the authors should add a row stating whether you measure the VAF of a mutation, or presence/absence of a mutation.

ONT recommendation is for flow cells that are run more than 10-12 hours each time. For, OCEANS, short 1-2 hour runs of flow cells are sufficient for variant calling. For such short runs, we have experimentally verified that the flow cells can be reused upto 10 times. We have included the missing costs for library preparation. Thanks for pointing this out.

10 - In general I find the comparison between NGS and ’NS’ in the introduction and in table 2 too opportunistic. The worst statistics are shown for NGS (e.g. not all machines take several days for sequencing), while for NS they mention uncommonly short sequencing times of fifteen minutes. Sure the main points are mostly correct, but it can be described a bit more realistic.

We have included a range for turnaround times to cover various scenarios for NGS and Nanopore Sequencing run times.

Reviewer 2:

In the presented manuscript a new protocol for accurate Nanopore Sequencing (NS) and subsequent detection of

somatic mutations with VAS limits of detection (LoD) between 0.05% and 1%.

The paper is very well written and easy to follow. The challenges for somatic SNP detection in low VAF NS data are clearly outlined, and a very detailed and comprehensive approach is taken to explain the novel protocol OCEANS, main features of which combine: * Stochastic Amplicon Ligation (SAL) which allows for creation of artificial longer fragments from shorter underlying DNA fragments, as to take advantage of the fact that NS platform throughput in the first hour of sequencing is mostly driven by the number of sequenced molecules, rather than their length * Blocker Displacement Amplification (BDA) which allow for enrichment of the mutation-burdened clone/allele in the analyzed tumor samples, which can be heterogeneous in nature, thus limiting the VAF for the mutation(s) of interest.

Comprehensive proof-of-concept testing of the proposed OCEANS protocol was carried, and the results are presented side-by-side the gold-standard NGS sequencing output. Proposed methodology shows comparative performance to the NGS results, while providing a sizable reduction in cost and turnover time, which are of great importance in clinical application settings.

While the paper, as I’ve mentioned before, is well structured and very nicely written, a brief outline of the paper structure at the end of the introduction section can benefit the reader as to provide guidance that, first, the SAL component of the protocol if described with the T+N data analyzed, and then the BDA part follows. It would also improve clarity if the explicit statement was made for the described SAL approach w.r.t. NS, as to whether this is suitable for any short-fragment library (e.g., FFPE data genome-wide, cfDNA, etc) or if some targeting is needed/recommended.

Thank you for the suggestion. We have included a brief outline in the introduction.

All illustration in the paper are of high quality and clearly demonstrate all the described concepts and/or results, providing a reader with a comprehensive visual aid.

Page 2: Some clarification on why the SAL-prepared longer fragments/reads resulted in better quality/error rate of NS will benefit the reader. Is this intrinsic to the sequencing process, or basecalling, or something else?

We have included the explanation for the better quality of SAL reads in the manuscript. This is mainly due to basecalling algorithm. According to ONT technical support, the lower quality score of shorter reads is due to lack of sufficient current signal information for proper normalization prior to basecalling by MinKNOW software.

Page 4: through the course of many PCR cycles - some quantification of ”many” is going to beneficial to the reader.

Thank you for the suggestion. We have included the quantification.

end of page 7, beginning of page 8: check VAR vs VRF correctness.

Thank you for pointing out the error. We have corrected the error.

Page 10: the mentioned limitation/non-suitability of cfDNA and FFPE samples for NS processing and subsequent detection of ”large-scale” alterations seems, from the outside, overoptimistic. Is there any reason why the proposed direct or SAL-based processing of FFPE and/or cfDNA can not be used for counting statistics detection, based on coverage profiles, of large-fragment, or even whole-chromosome CNV events? If not – the reader will benefit from learning why. If possible, even in theory, this can be of tremendous interest to the community, both academic and clinical, as the turnaround time with NS is much better than with NGS.

The limitation of FFPE and cfDNA for nanopre sequencing is the short length profile of the DNA in those samples. The short length of the original sample makes them unsuitable to utilize the long-read capabilities of nanopore sequencing without a concatenation step like SAL. We mentioned this in the discussion section.

Page 10: there may be a benefit of adding to the discussion mentioned about most recent progress of ONT small variant calling methodologies PEPPER/DeepVariant, and how they may improve the specificity/sensitivity of the proposed protocol, or if they are not really suitable for this application.

Thank you for the suggestion. We have included a discussion on the variant calling methodologies in the discussion section.

Overall, this is a very nicely written paper with a great science behind it. I strongly recommend this paper for publication after a minor revision, which would be better referred to as ”polishing”.

Minor comments: * page 1, introduction: (VAF) remaining challenging - (VAF) remains Thanks for pointing out the error. We have corrected the error.

* page 6, Fig 4, panels c and e: either add (5) to Y axis label, or to Y axis values.
Thanks for pointing out the error. We have corrected the error.

Thank you for your careful consideration and evaluation of this work.

**Second round of review**

**Reviewer 1**

The authors have substantially improved their manuscript. My remaining concerns are as follows:

I appreciate the addition of the precision recall curves, as they are more informative. However, in figure 5 it remains unclear how the N=110 mutations are selected (also holds true for fig 6 g and h). This does not seem to be in line with those presented in Fig 5c.

The ROC curves, in my opinion, don't add anything (and may even be a bit misleading as they report 99.99% AUC, which - while true - is not very informative given the large number of negatives. Their only purpose is to demonstrate that, similar to NGS, the bulk of the (negative) loci are not called (i.e. are true negative). This is already abundantly clear from Fig 5c, and could be simply summarized by stating that at 100% sensitivity there is a very low FP rate (or a very high TN rate).

In Figure 5d, only the ddPCR results which were positive are shown. Why not also include the ddPCR negative positions with a different color from table 1?

The authors write in the discussion: "We underestimated the sample VAF if the VRF saturates or if the Nanopore error rate at that particular mutation position was high". Especially the latter (error rate) is not apparent from the data. Could the high-error positions be indicated with a color in Figure 5d, similar to saturated VRF locations?

Thank you for providing the customized python code in a github repository “snippy.” With the example command line, I expect to be able to reproduce most of the results. However, the Supplementary Excel Table (mentioned in Availability of data and materials) is missing in which the reads mapped to MUT and WT alleles are reported.

For the results to be reproducible, a list of read names that remained after the downsampling needs to be provided. There are two downsampling steps in the pipeline. It would be advised to provide a short description of why these downsamplings are necessary.

The OCEANS Oligo List (excel) is missing from the new version of supplementary materials.

**Authors Response**

**Point-by-point responses to the reviewers’ comments:**

The authors have substantially improved their manuscript. My remaining concerns are as follows:

I appreciate the addition of the precision recall curves, as they are more informative. However, in figure 5 it remains unclear how the N=110 mutations are selected (also holds true for fig 6 g and h). This does not seem to be in line with those presented in Fig 5c.

*Response: Thank you for your comment. We calculated the precision recall curves for 110 amino acid mutations from the 114 nucleotide loci mutations called by Clair in Fig. 5c. Since, some amino acid mutations involved two nucleotide change like BRAF(c.1798 1799delinsAA p.V600K) the number of nucleotide loci mutations and the amino acid mutations are not the same. We included this explanation in the figure legends.*

The ROC curves, in my opinion, don't add anything (and may even be a bit misleading as they report 99.99% AUC, which - while true - is not very informative given the large number of negatives. Their only purpose is to demonstrate that, similar to NGS, the bulk of the (negative) loci are not called (i.e. are true negative). This is already abundantly clear from Fig 5c, and could be simply summarized by stating that at 100% sensitivity there is a very low FP rate (or a very high TN rate).

*Response: Thank you for your comment. We removed the ROC curves from the figures*.

In Figure 5d, only the ddPCR results which were positive are shown. Why not also include the ddPCR negative positions with a different color from table 1?

*Response: Thank you for your suggestion. We included the ddPCR negative position in Fig 5d.*

The authors write in the discussion: "We underestimated the sample VAF if the VRF saturates or if the Nanopore error rate at that particular mutation position was high". Especially the latter (error rate) is not apparent from the data. Could the high-error positions be indicated with a color in Figure 5d, similar to saturated VRF locations?

*Response: Thank you for your suggestion. The higher error positions were observed only in the NSCLC and HCC panels. We indicated these positions in Fig 6C and Fig 6D.*

Thank you for providing the customized python code in a github repository “snippy.” With the example command line, I expect to be able to reproduce most of the results. However, the Supplementary Excel Table (mentioned in Availability of data and materials) is missing in which the reads mapped to MUT and WT alleles are reported.

*Response: We included the supplementary excel file in our previous revision. The file could be missing due to technical issues. We are also including this file in the current revised version as additional file 2.*

For the results to be reproducible, a list of read names that remained after the downsampling needs to be provided. There are two downsampling steps in the pipeline. It would be advised to provide a short description of why these downsamplings are necessary.

*Response: Thank you for pointing out this missing information. We included the subsampled read names in a separate text file - Additional file 5. We also included the reason for subsampling in the methods section.*

The OCEANS Oligo List (excel) is missing from the new version of supplementary materials.

*Response: We included the supplementary excel file in our previous revision. The file could be missing due to technical issues. We are also including this file in the current revised version as additional file 3.*

*Thank you for your careful consideration and evaluation of this work.*
